# *SEPALLATA­*-like genes of *Isatis indigotica* can affect the architecture of the inflorescences and the development of the floral organs

**DOI:** 10.7717/peerj.13034

**Published:** 2022-03-01

**Authors:** Yan-Qin Ma, Zuo-Qian Pu, Xiao-Min Tan, Qi Meng, Kai-Li Zhang, Liu Yang, Ye-Ye Ma, Xuan Huang, Zi-Qin Xu

**Affiliations:** College of Life Sciences, Northwest University, Xi’an, Shaanxi, China

**Keywords:** *Isatis indigotica* Fortune, *Arabidopsis thaliana*, *SEPALLATA*-like genes, Inflorescence architecture, Floral transition, Floral organ differentiation, Petal rescue, Floral meristem determinacy, Overexpression, Floral homeotic conversion

## Abstract

**Background:**

The architecture of inflorescence and the development of floral organs can influence the yield of seeds and have a significant impact on plant propagation. E-class floral homeotic MADS-box genes exhibit important roles in regulation of floral transition and differentiation of floral organs. Woad (*Isatis indigotica*) possesses unique inflorescence, floral organs and fruit. However, very little research has been carried out to determine the function of MADS-box genes in this medicinal cruciferous plant species.

**Results:**

*SEPALLATA* orthologs in *I. indigotica* were cloned by degenerate PCR. The sequence possessing the highest identity with *SEP2* and *SEP4* of Arabidopsis were named as *IiSEP2* and *IiSEP4*, respectively. Constitutive expression of *IiSEP2* in Columbia (Col-0) ecotype of Arabidopsis led to early flowering, and the number of the flowers and the lateral branches was reduced, indicating an alteration in architecture of the inflorescences. Moreover, the number of the floral organs was declined, the sepals were turned into carpelloid tissues bearing stigmatic papillae and ovules, and secondary flower could be produced in apetalous terminal flowers. In *35S::IiSEP4-GFP* transgenic Arabidopsis plants in Landsberg *erecta* (L*er*) genetic background, the number of the floral organs was decreased, sepals were converted into curly carpelloid structures, accompanied by generation of ovules. Simultaneously, the size of petals, stamens and siliques was diminished. In *35S::IiSEP4-GFP* transgenic plants of apetalous *ap1 cal* double mutant in L*er* genetic background, the cauliflower phenotype was attenuated significantly, and the petal formation could be rescued. Occasionally, chimeric organs composed of petaloid and sepaloid tissues, or petaloid and stamineous tissues, were produced in *IiSEP4* transgenic plants of *apl cal* double mutant. It suggested that overexpression of *IiSEP4* could restore the capacity in petal differentiation. Silencing of *IiSEP4* by Virus-Induced Gene Silencing (VIGS) can delay the flowering time, and reduce the number and size of the floral organs in woad flowers.

**Conclusion:**

All the results showed that *SEPALLATA*-like genes could influence the architecture of the inflorescence and the determinacy of the floral meristems, and was also related to development of the floral organs.

## Introduction

The changing process of plant growth from vegetative stage to reproductive stage is known as floral transition and is associated with highly complicated regulatory networks. In the initial period of floral transition, the shoot apical meristem will expand in diameter and become inflorescence meristem gradually. In Arabidopsis, floral meristems will form continuously on the flank of inflorescence meristem and arrange spirally along the stem. In the course of subsequent growth, primordia of four floral organ types will appear in specific areas of floral meristems from outside to inside, and they will differentiate into sepals, petals, stamens and pistil, respectively (Smyth, Bowman & Meyerowitz, 1990).

MADS-box family genes play crucial roles in reproduction of flowering plants (Ma et al., 2020). In 1990’s, Coen and Meyerowitz proposed the “ABC model” for floral organ construction, which provided a theoretical basis for elucidation of Gothe’s intuitive idea that all floral organs have arisen from a modified leaf (Coen & Meyerowitz, 1991). The flowers in most angiosperms are composed of four whorls of floral organs: sepals, petals, stamens and carpels. The identity of these floral organs is determined by a specific combination of floral homeotic MADS-box transcriptional factors, as suggested in “floral quartet model” and ABCDE model (Pelaz et al., 2001; Honma & Goto, 2001; Theissen & Saedler, 2001; Shen et al., 2019). In the floral quartet model, complexes of MADS-box proteins are supposed to be involved in specifying the identity of different floral organs (Theissen & Saedler, 2001; Gramzow & Theissen, 2010).

In Arabidopsis, there are four E-class floral homeotic genes and they are named as *SEPALLATA1* (*SEP1*, previously known as *AGL2*), *SEP2* (*AGL4*), *SEP3* (*AGL9*), and *SEP4* (*AGL3*). Mutation in anyone of these *SEP* genes produces only subtle phenotypic changes, implying their functional redundancy (Pelaz et al., 2000). Consistent with this, the four different types of floral organs in *sep1 sep2 sep3* triple mutant are converted into sepals, accompanied by loss of flower determinacy. More seriously, the flowers of *sep1 sep2 sep3 sep4* quadruple mutant are comprised entirely of leaf-like structures (Pelaz et al., 2000).

Nonetheless, *SEP1*, *SEP2* and *SEP3* are not completely redundant with *SEP4*, owing to the different phenotypes between the triple mutant and the quadruple mutant. Further investigation revealed that *SEP4* had a more prominent role in maintenance of the determinacy of floral meristems, because *ap1 sep4* double mutants display a cauliflower-like phenotype which was observed neither in the respective single mutants nor in *ap1 sep1 sep2* or *ap1 sep3* mutants (Ditta et al., 2004). The single mutants of *SEP3* produced sepaloid petals resemble to those of the intermediate *ap1* mutants (Pelaz et al., 2001), indicating the redundancy of *SEP1*, *SEP2* and *SEP3* is not complete either. Besides, the dramatic floral defects of *lfy sep3* double mutants are not found in either of the single mutants (Liu et al., 2009). It suggests that the phenotypic variations produced by loss-of-function allele of *SEP3* could not be compensated by other *SEP* genes when *LFY* was mutagenized simultaneously.

The functional differences among *SEP* genes are probably associated with their inconsistency in expression. The transcripts of *SEP1*, *SEP2* and *SEP4* are detectable in floral meristems and in sepal primordia. In contrast, *SEP3* is activated at the end of the second stage during flower development (Flanagan & Ma, 1994; Savidge, Rounsley & Yanofsky, 1995; Mandel & Yanofsky, 1998). Unlike other *SEP* genes, the transcription of *SEP4* is not confined to flowers, the mRNA of this gene can also be detected in vegetative tissues, including leaves and stems (Ma, Yanofsky & Meyerowitz, 1991). It is very likely that these variations in expression are related to the diversified functions of the *SEP* genes (Jack, 2001).

A number of *SEP*-like genes have been characterized in other plant species. Down-regulation of *GRCD1* (*Gerbera Regulator of Capitulum Development 1*), a homologous gene of *SEP1* in *Gerbera hybrida*, converted the female florets into petals (Kotilainen et al., 2000). Inhibition of *TM29*, a tomato *SEPALLATA* homolog, resulted in aberrant phenotypes in the inner whorls of floral organs, resembling the cosuppression lines of *FBP2* in petunia and *TM5* in tomato, two other *SEP*-like genes (Ampomah-Dwamena et al., 2002). In cultivar ‘Ryokusei’ of *Habenaria radiate*, a mutant containing a retrotransposon-like element in the first exon of *HrSEP-1*, the white petals were changed into greenish petals, sepaloid tissues and a ventral column were formed in the inner whorls of flowers. Transcript of *HrSEP-1* was undetectable in ‘Ryokusei’, namely loss-of-function of *HrSEP-1* could influence the development of the floral organs (Mitoma & Kanno, 2018).

In five *SEP*-like genes of London plane (*Platanus acerifolia*), a basal eudicot tree, *PlacSEP1.1*, *PlacSEP1.2* and *PlacSEP1.3* belong to the *SEP1/2/4* clade, *PlacSEP3.1* and *PlacSEP3.2* are members of *SEP3* clade. The transcripts of *PlacSEP1.3*, *PlacSEP3.1* and *PlacSEP3.2* were detectable during the initiation stage of male and female inflorescences, and throughout the development of flowers and fruits, whereas *PlacSEP1.2* was expressed only in female inflorescences. In addition, the expression of *PlacSEP1.3* and *PlacSEP3.1* was weak in vegetative buds of adult trees, implying their possible roles in regulation of dormancy. These results also showed that patterning of the inflorescences was associated with *SEP*-like genes. Except in cases of early flowering, overexpressing tobacco plants of *PlacSEP1.1* and *PlacSEP3.1* produced more lateral branches (Zhang et al., 2017a). Knockdown of *SLMBP21*, a tomato *SEPALLATA*-like gene in FBP9/23 subclade, could suppress the development of abscission zone in flower pedicels by affecting a subset of genes related to meristem activity, and overexpression of this gene could produce small cells at the proximal section of pedicels and peduncles (Liu et al., 2014).

*Thalictrum thalictroides* is a non-core eudicot species whose flowers are apetalous. By Virus-Induced Gene Silencing (VIGS), *SEP*-like genes in *T. thalictroides* were found to be partially redundant in specifying the identity of sepals and stamens, and in promoting petaloidy of sepals. Moreover, the ortholog of *SEP3* showed a pronounced role in determination of carpel identity, and in carpel development. Furthermore, *ThtSEP1* was involved in defining the boundary between sepals and stamens, and *ThtSEP2* functioned in maintaining the boundary between stamens and pistil. In double-knockdown or triple-knockdown plants, partial to complete homeotic conversion of stamens and carpels to sepaloid organs or green sepals was occurred (Soza et al., 2016). Silencing of *FaMADS9*, a *SEP1/2*-like gene in strawberry, could inhibit the development of petals, and the ripening of achene and receptacle (Seymour et al., 2011).

In cucumber (*Cucumis sativus* L.), a mutant showing perturbations in the development of stamens, pistils and fruits was found to be related to *CsSEP2*. In this *SEP*-like gene, the change at the donor splice site in 5’-end of the sixth intron results in skipping of the sixth exon during RNA processing, which leads to loss of the transcriptional activation capacity of the protein encoded by the alternative transcript. The phenotypes of *CsSEP2* mutant include enormously large sepals in female and male flowers, irregular shape or loss of three stigma lobes and three-fold longer style in female flowers, larger male flowers with separate filaments and an inner cavity filled by style-like tissues. Moreover, elongated carpel primordia can be observed before anthesis in female and male flowers of the mutant, accompanied by early shedding of the fruits (Wang et al., 2016). These results indicated that *CsSEP2* was a homologous gene of Arabidopsis *SEP2* and was involved in floral organ and fruit development in cucumber.

Woad (*Isatis indigotica* Fortune) is one of the traditional Chinese medicinal plants and is a biennial crucifer with bisexual flowers. Woad flower is composed of four whorls of floral organs: sepals, petals, stamens, and carpels. Up to now, a good deal of research has been devoted to examine the medicinal value of woad (Hu et al., 2011; Li et al., 2014), but the genes involved in regulation of flowering in this plant species are so far very rarely concerned. There are also four *SEP*-like genes in woad, and these genes were named as *IiSEP1*, *IiSEP2*, *IiSEP3* and *IiSEP4* according to the degree of similarity with the corresponding orthologous gene in Arabidopsis (Ma et al., 2019; Pu et al., 2020). In the present work, the function of *IiSEP2* and *IiSEP4* was investigated. To gain insight into the conservation of *SEP* activity among different species, transgenic Col-0 (Columbia) Arabidopsis plants overexpressing *IiSEP2* were generated and analyzed. *IiSEP4* isolated in previous work of our laboratory (Pu et al., 2020) was used in preparation of the ectopic expressing lines of wild-type Arabidopsis or *ap1 cal* double mutant in L*er* (Landsberg *erecta*) genetic background. At the same time, silencing by VIGS was carried out to determine the function of *IiSEP4*.

## Materials and Methods

### Plant materials and bacterial strains

Woad plants, wild-type Arabidopsis ecotype Col-0, wild-type Arabidopsis ecotype L*er*, and *ap1 cal* double mutant in L*er* genetic background (CS67157) were used as materials in the present work, and were grown under normal greenhouse conditions (23 ± 2 °C, 16 h light/8 h dark). Woad flowers and leaves were used as materials in extraction of RNA and DNA. pMD 18-T was used in cloning of cDNA and genomic DNA fragments. pCAMBIA1302 was used in construction of the binary expression vectors. *Escherichia coli* strain DH5α and *Agrobacterium tumefaciens* strain GV3101 were used as host in isolation of *IiSEP2* and in transformation of Arabidopsis, respectively.

### Cloning of the coding sequence and the promoter of *IiSEP2*

Total RNA was extracted from floral organs of *I. indigotica* with Trizol reagent (Invitrogen, Waltham, MA, USA). Genomic DNA was eliminated by RNase-free DNase I (Takara, Tokyo, Japan). First strand cDNA was synthesized with PrimeScript 1st Strand cDNA Synthesis Kit (Takara, Tokyo, Japan). The coding sequence of *IiSEP2* was obtained by PCR and the primers were designed according to the open reading frames of the MADS-box genes in different plant species (Table S1).

Promoter sequences were isolated with Universal Genome Walker 2.0 Kit (Clontech, Mountain View, CA, USA). The primers were designed according to the sequence in the first exon of *IiSEP2* (Table S1). Genomic DNA was extracted from leaves of *I. indigotica* by CTAB method (Stewart & Via, 1993). Uncloned libraries were created after restriction digestion of the genomic DNA with *Dra* I, *Eco*R V, *Pvu* II or *Stu* I, and ligation of the adaptors provided by Universal Genome Walker 2.0 Kit (Clontech, Mountain View, CA, USA). Upstream sequences were amplified with Adaptor Primer 1 and IiSEP2-Upstream1 with Advantage 2 PCR Kit (Clontech, Mountain View, CA, USA). Nested PCR was carried out with Adaptor Primer 2 and IiSEP2-Upstream2. The PCR products were ligated into pMD 18-T and were sequenced after identification by colony PCR.

### Analyses of the expression pattern of *IiSEP 2* in *I. indigotica*

Total RNA was extracted from different tissues and floral organs of *I. indigotica* with RNAprep Pure Plant Kit (TIANGEN, Beijing, China) and the first strand cDNA was synthesized with PrimeScript 1st Strand cDNA Synthesis Kit (Takara, Tokyo, Japan). Quantitative real-time PCR (qRT-PCR) was conducted in BIO-RAD real-time PCR system (BIO-RAD, Hercules, CA, USA) with the primers shown in Table S1 (Huang et al., 2008; Ma et al., 2019). Woad *actin* (GenBank accession No. AY870652.1) was used as a reference gene. Triplicate qRT-PCRs were carried out and relative quantification of the transcript levels was accomplished using the comparative threshold cycle (Ct) method. Relative quantification refers to that the PCR signals of the *IiSEP2* transcript in leaves, stem apexes, early inflorescences (the inflorescence shrinks into a ball), later inflorescences (the inflorescence is completely unfolded), flowers in full bloom, floral organs (include sepals, petals, stamens, and pistils), and young silicles are normalized to the PCR signal in roots. The fold change was calculated using the following formula: fold change = 2^−∆∆Ct^, where ∆∆Ct = (Ct_*IiSEP2*_ − Ct_*Iiactin*_) _different sample_ − (Ct_*IiSEP2*_ − Ct_*Iiactin*_) _root_.

### Subcellular localization by confocal laser scanning microscopy

The coding sequence of *IiSEP2* was inserted into pCAMBIA1302 containing CaMV 35S promoter after high-fidelity amplification with the primers shown in Table S1. The recombinant construct was introduced into competent cells of *A. tumefaciens* GV3101 by freeze-thaw method (Höfgen & Willmitzer, 1988). Transformed *A. tumefaciens* cells were selected on YEB medium containing rifampicin (50 μg/L), gentamicin (50 μg/L), and kanamycin sulfate (50 μg/L). Positive colony carrying recombinant plasmid was propagated in liquid medium for 24 h at 30 °C in a shaker incubator (~200 rpm). Precipitated bacterial cells were resuspended with fresh YEB medium containing the same antibiotics and were grown to an OD_600_ value of 0.8. The bacterial cells were collected by centrifugation at 6,000 rpm for 10 min under room temperature. The pellet was resuspended with agroinfiltration buffer (10 mmol/L 2-(*N*-morpholino)ethanesulfonic acid, 200 μmol/L acetosyringone, 10 mmol/L MgCl_2_) to an OD_600_ value of 0.7. The bacterial suspension was infiltrated into the abaxial leaf epidermis of 6-week-old *Nicotiana benthamiana* plants using a syringe without needle. The quality of transient expression was estimated 60 h after agroinfiltration. Confocal laser scanning microscopy of the living plant tissues was performed using an OLYMPUS FLUOVIEW FV1000 microscope. Green fluorescence of GFP was observed with an exciting light of 488 nm.

### Identification of *IiSEP2* transgenic Arabidopsis plants

The recombinant binary expression vector pCAMBIA1302-*IiSEP2*, in which *IiSEP2* is controlled by CaMV 35S promoter, was introduced into *A. tumefaciens* GV3101. *Agrobacterium*-mediated transformation of Arabidopsis was performed essentially according to the protocol of Clough & Bent (1998). Col-0 transgenic seedlings were selected on hygromycin-containing medium, and the expression levels of *IiSEP2* in T2 generation were detected by RT-PCR using *IiSEP2* specific primers (Table S1), and by Western blotting using rabbit monoclonal antibody of GFP (Sino Biological Inc., Beijing, China). Transgenic phenotypes were confirmed in T1 and T2 generations. Influence of *IiSEP2* on Arabidopsis MADS-box genes, including *AP3* (*APETALA3*), *PI* (*PISTILLATA*), *SHP1* (*SHATTERPROOF1*) and *SHP2*, was determined by qRT-PCR.

### Preparation of *IiSEP4* transgenic plants in the genetic background of L*er*

The binary expression vector pCAMBIA1302-*IiSEP4* constructed in our previous work (Pu et al., 2020) was introduced into *A. tumefaciens* GV3101 and was used in genetic transformation of wild-type Arabidopsis plants (L*er*) or *ap1 cal* double mutant (L*er*). The expression of the transgene was analyzed by qRT-PCR with IiSEP4-qRT-F (5′-AGATAGCCGGGATGGGAGTG-3′) and IiSEP4-qRT-R (5′-AATCATCGACCGGGCCTTTG-3′) (Pu et al., 2020). To compare the phenotypic differences, 25 *IiSEP4* transgenic Arabidopsis plants were grown in greenhouse together with 25 wild-type plants every time.

### Silencing of *IiSEP4* by VIGS

To investigate the function of *IiSEP4* in woad, its expression was downregulated by *Agrobacterium*-mediated and TRV-based (tobacco rattle virus-based) VIGS (Liu, Schiff & Dinesh-Kumar, 2002). *A. tumefaciens* GV3101 was cultured in LB liquid medium containing 50 mg/L kanamycin, 25 mg/L rifampicin and 25 mg/L gentamicin to OD_600_ = 2.0. After centrifugation, the bacterial cells were resuspended with infiltration solution (100 mL was composed of 100 µL 0.2 mol/L acetosyringone, 2 mL 0.5 mol/L 2-(*N*-morpholino)ethanesulfonic acid, 1 mL 1 mol/L MgCl_2_). During four-leaf stage of growth, the leaves of woad plants were infiltrated with a mix of *A. tumefaciens* GV3101 carrying pTRV1 and *A. tumefaciens* GV3101 carrying recombinant pTRV2-IiSEP4 containing a 300 bp specific fragment coming from 5’-end of *IiSEP4* coding sequence (Pu et al., 2020). The fragment of *IiSEP4* was amplified by high-fidelity PCR with primers VIGS-IiSEP4-F (5′-CGCGAATTCCGATGATTGATCAACTATCG-3′) and VIGS-IiSEP4-R (5′-CGCGGATCCTCTGGGATGTTGTTGCAGAG-3′). In each VIGS experiment, the phenotypes of 30 woad plants in treatment group and 30 woad plants in control group were compared.

### Data analysis

SPSS 22.0 (SPSS Inc., Chicago, IL, USA) was used for data analysis. The data of rosette leaf number, the angle between cauline leaf and stem, flower bud number, stamen number in *IiSEP2* overexpressing Arabidopsis plants, and the data about the expression pattern of *IiSPE2* in woad plants, were analyzed by one-way ANOVA, followed by Tukey’s post-hoc test. The data obtained in qRT-PCR analysis of the MADS-box genes (including *AP3*, *PI*, *SHP1* and *SHP2*) in *IiSEP2* overexpressing Arabidopsis plants, *IiSEP4* in 35S::*IiSEP4-GFP* transgenic plants in wild-type L*er* genetic background and in 35S::*IiSEP4-GFP* transgenic plants of *ap1 cal* double mutant in L*er* genetic background, and *IiSEP4* in distal noninfiltrated leaves in woad plants infiltrated with pTRV1 + pTRV2-IiSEP4, were analyzed by two-sided Student’s *t*-test (Pu et al., 2020).

## Results

### Identification of the coding sequence and the promoter of *IiSEP2*

The coding sequence of *IiSEP2* was amplified with high-fidelity DNA polymerase by RT-PCR. Sequencing result showed that the open reading frame of *IiSEP2* was 753 bp in length and encoded a 250-amino-acid protein (Fig. S1). IiSEP2 comprises the four regions of typical plant MIKC-type MADS-box proteins, namely MADS domain (M, 2-60aa), intervening region (I, 61-83aa), keratin-like domain (K, 84-171aa), and C-terminal region (C, 172-250aa). At the same time, the protein encoded by *IiSEP2* shows high identity in amino acid sequence with the homologous proteins of other plant species. For instance, the identity between IiSEP2 and Arabidopsis SEP2 is 95% (Fig. S2A). Eight conserved motifs with a length of 6~50 amino acids could be found in IiSEP1, IiSEP2 and IiSEP2-like proteins by the online tool in MEME website (http://meme-suite.org/) (Bailey et al., 2009), and two motifs (Motif 1 and Motif 9) in MADS domain and three motifs (Motif 3, Motif 4 and Motif 5) in K-box domain existed in all the analyzed proteins (Fig. S2B). The logo of these motifs discovered by MEME is shown in Fig. S2C.

To clarify the relationship of IiSEP2 and other plant MADS-box proteins, we analyzed the phyletic evolution of IiSEP2 (Fig. S3). The results showed that IiSEP2 was closely related to Arabidopsis SEP2. These results demonstrated that *IiSEP2* was an E-class floral homeotic gene and further confirmed that *IiSEP2* was the orthologous gene of Arabidopsis *SEP2*.

In 1 kb regulatory sequence upstream of the initiation codon characterized by Genome Walking method (Sequence Data S1), there are three CArG-boxes, indicating the expression of *IiSEP2* can be affected by other MADS-box transcriptional factors.

### Spatial and temporal expression of *IiSEP 2* in *I. indigotica*

**Figure 1:**
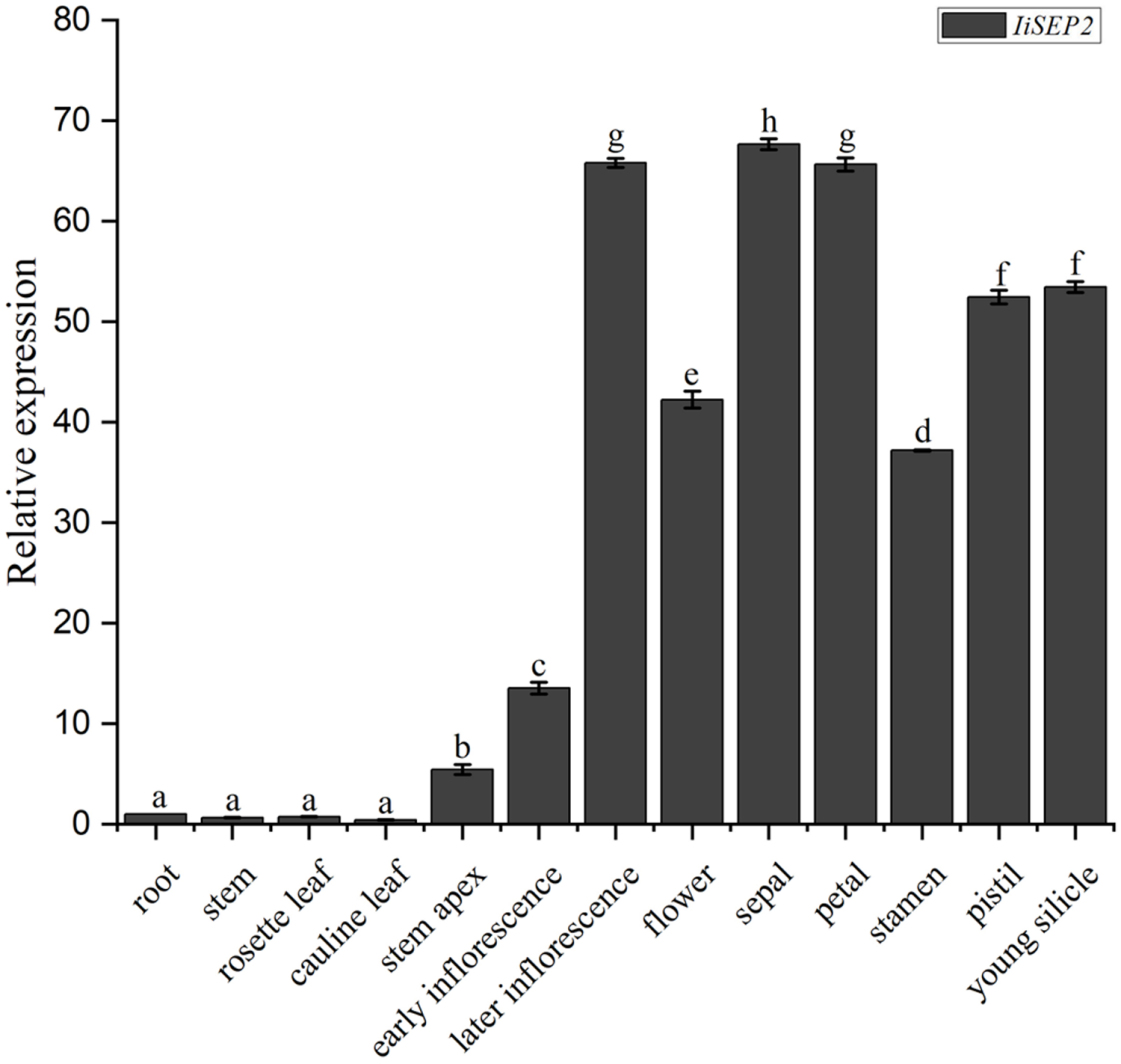
Expression pattern of *IiSEP2* in *I. indigotica* analyzed by qRT-PCR. Error bars represent the standard deviation. Root was used as normalization sample. Multiple testing correction was conducted with SPSS software, different letters indicate significant difference at 5% level.

The abundances of *IiSEP2* mRNA in various tissues and floral organs of *I. indigotica* were analyzed by qRT-PCR. The transcriptional levels of *IiSEP2* were low in roots and leaves. However, the quantity of *IiSEP2* transcript was significantly increased in stem apex and was accumulated to a high level in flowers and young silicles. Further testing in floral organs showed that *IiSEP2* mRNA was highly expressed in sepals, petals, stamens, and pistil (Fig. 1). Taken together, the qRT-PCR results illustrate that *IiSEP2* expression occurs from the early stages of flower development to young silicles.

### Subcellular localization of IiSEP2

To determine the subcellular localization of IiSEP2 *in vivo*, the coding sequence of *IiSEP2* without the stop codon was fused in frame with *GFP* reporter gene in pCAMBIA1302 and was driven by CaMV 35S promoter. *A. tumefaciens* carrying the recombinant expression vector was used to perform a transient expression assay in *N. benthamiana* leaves. The GFP fluorescence of the control experiment using empty vector evenly distributed throughout the cell, whereas the GFP fluorescence of IiSEP2-GFP was observed only in the nucleus (Fig. S4). The result is consistent with the predictions using PSORT II online server (http://psort.hgc.jp/form2.html), namely KRIENKINRQVTFAKRR in N-terminus of IiSEP2 is a bipartite nuclear localization signal (NLS).

### Overexpression of *IiSEP2* leads to morphologic changes in transgenic Arabidopsis plants

To examine the function of *IiSEP2*, transgenic Arabidopsis plants under the control of the constitutive CaMV 35S promoter were prepared in this work. Seeds were selected on MS medium containing hygromycin, and the putative transgenic plants were confirmed by PCR and Western blotting (Fig. S5).

**Figure 2:**
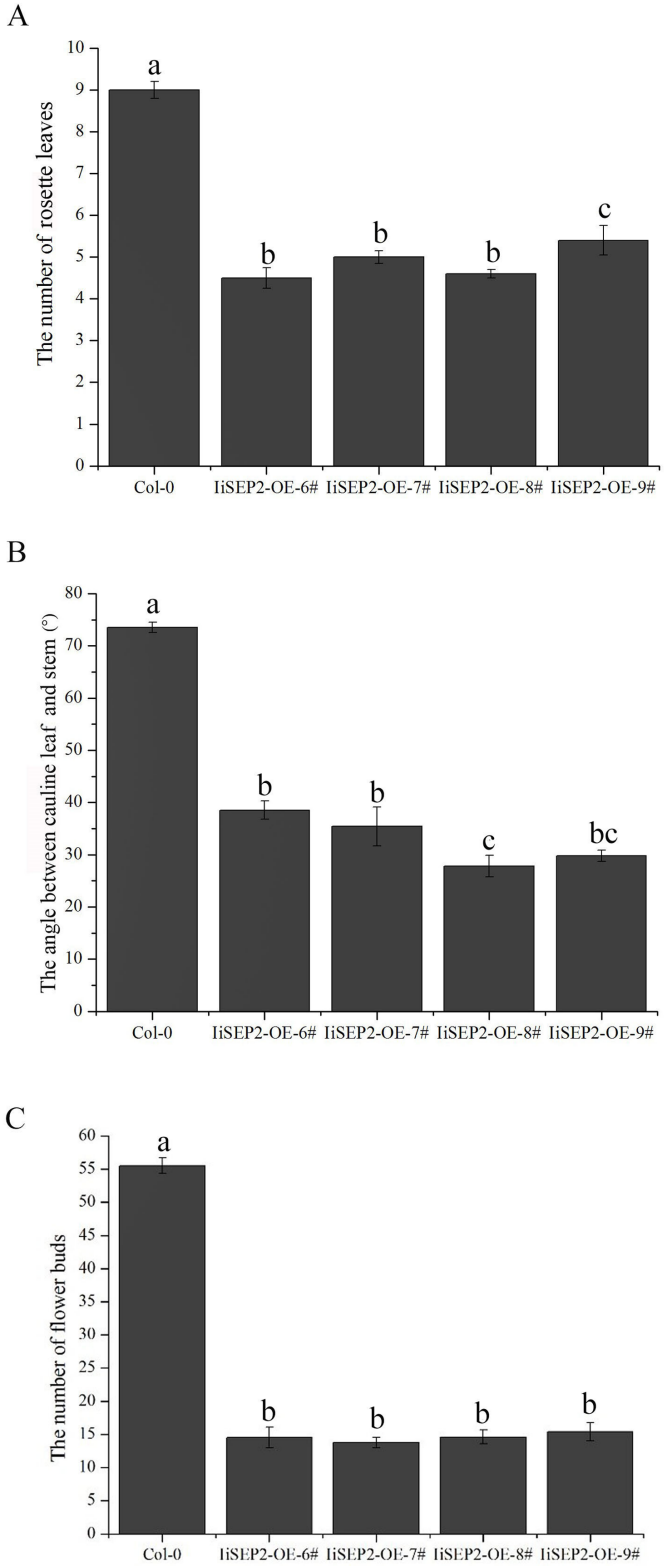
Effects of *IiSEP2* overexpression on aerial architecture of Arabidopsis plants. (A) Comparison of rosette leaf number produced by *35S::IiSEP2* transgenic and wild-type Col-0 plants before flowering. (B) Comparison of the angle between the cauline leaves and the stems. (C) Comparison of the flower bud number produced by *35S::IiSEP2* transgenic and wild-type Col-0 plants. Values correspond to mean ± standard error (*n* = 15). Multiple testing correction was conducted with SPSS software; different letters indicate significant difference at 5% level.

In 28 *35S::IiSEP2* independent transgenic lines, nine transgenic lines showed obvious macroscopic differences in comparison with the wild-type Arabidopsis plants. *35S::IiSEP2* transgenic Arabidopsis plants were extremely early in bolting. The flowering time was evaluated by counting the number of the leaves produced when the first flower bloomed. The transgenic lines generated 4–5 leaves prior to the onset of flowering, whereas wild-type plants produced approximately nine leaves (Fig. 2A).

**Figure 3:**
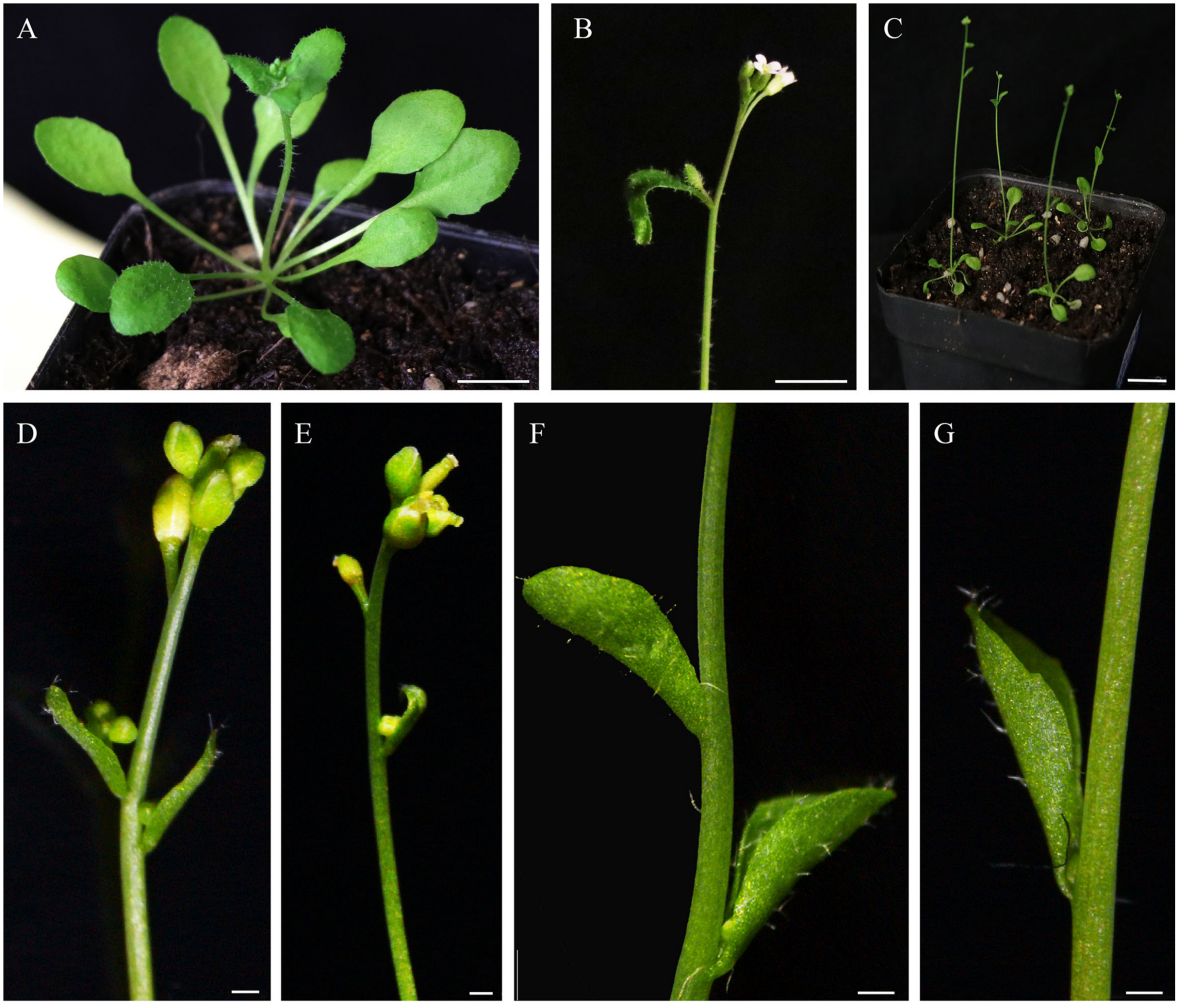
Morphological comparison of the cauline leaves between wild-type Col-0 and *IiSEP2* transgenic plants. (A) The rosette leaves and cauline leaves of wild-type Col-0 plants, the angles between the oval-shaped cauline leaves and the stems are larger. (B) The upper part of a wild-type Col-0 plant in the flowering stage. (C) *IiSEP2* transgenic Arabidopsis plants. (D) IiSEP2-OE-6#. (E) IiSEP2-OE-7#. (F) IiSEP2-OE-8#. (G) IiSEP2-OE-9#. *IiSEP2* transgenic Arabidopsis plants in (F) and (G) show the inward-curling cauline leaves, and the angles between the cauline leaves and the stems are reduced. In (A–C), scale bars represent 1 cm. In (D–G), scale bars represent 1 mm.

In wild-type Col-0 Arabidopsis plants, cauline leaves are generated at the lower part of the inflorescence stems, and the oval-shaped blades are slightly curved outward. Moreover, the angles between the cauline leaves and the stems in wild-type Col-0 Arabidopsis plants are larger (Figs. 2B, 3A and 3B). Different from wild-type Col-0 Arabidopsis plants, the cauline leaves of *IiSEP2* transgenic Arabidopsis plants are clearly curling inward and the margin is involute. Simultaneously, the angles between the cauline leaves and the stems become smaller in transgenic Arabidopsis plants, and the whole blade looks like an inverted cone (Figs. 2B and 3C–3G).

**Figure 4:**
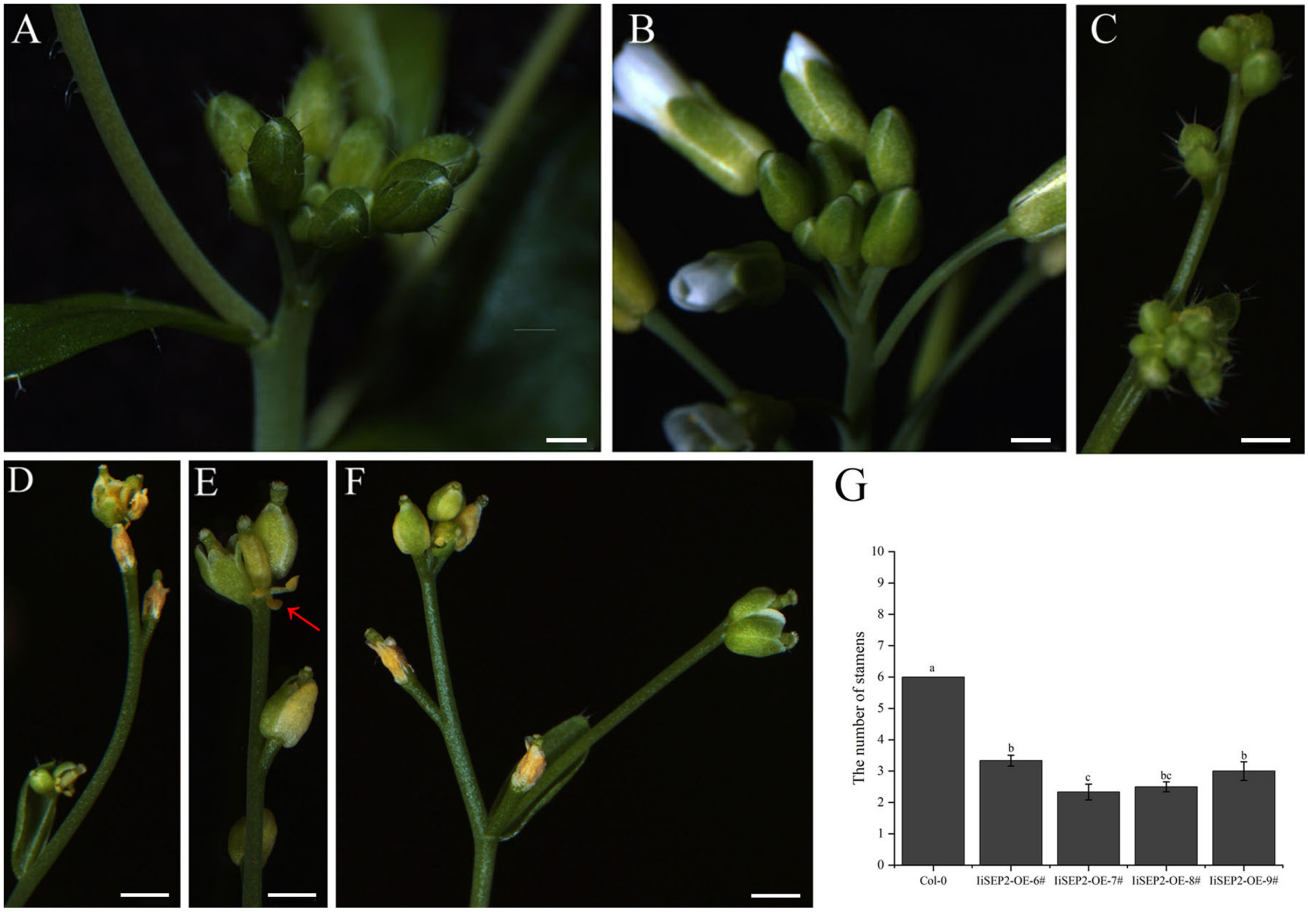
Influence of *IiSEP2* on development of inflorescences. (A & B) Indefinite inflorescences of wild-type Col-0 plants. The inflorescence meristem could produce new flower buds continuously. (C) Inflorescence of *IiSEP2* overexpressing Arabidopsis plant, only a few flower buds could be observed. (D) Wilted inflorescence with undersized flower buds at early development stage in *IiSEP2* transgenic Arabidopsis plant. (E & F) The inflorescences converted into defective terminal flowers in *IiSEP2* transgenic Arabidopsis plant, arrow indicate stamens. (G) Comparison of the number of stamens per flower. Scale bars represent 1 mm. Multiple testing correction was conducted with SPSS software, different letters indicate significant difference at 5% level.

The process that the plant switches from vegetative growth to reproductive growth is named as floral transition. At the early stage of floral transition, the vegetative shoot apical meristem is transformed into an inflorescence meristem, and floral meristems are formed in the peripheral regions of the inflorescence meristem. Then the primordia of the floral organs will be produced along with the development of floral meristems, which is accompanied by definition of the boundaries between different floral organs. The inflorescence meristem of wild-type Col-0 Arabidopsis plants is highly active during the growth process and can produce a lot of flower buds continuously (Figs. 4A and 4B). However, the activity of the inflorescence meristem in *IiSEP2* transgenic Arabidopsis plants is reduced remarkably. In general, only a few flower buds could be generated at the top of the inflorescence stem in *IiSEP2* transgenic Arabidopsis plants (Figs. 2C and 4C–4F), illustrating that constitutive expression of *IiSEP2* could reduce the number of flowers and the number of lateral branches. Namely, IiSEP2 can affect the architecture of the inflorescences.

**Figure 5:**
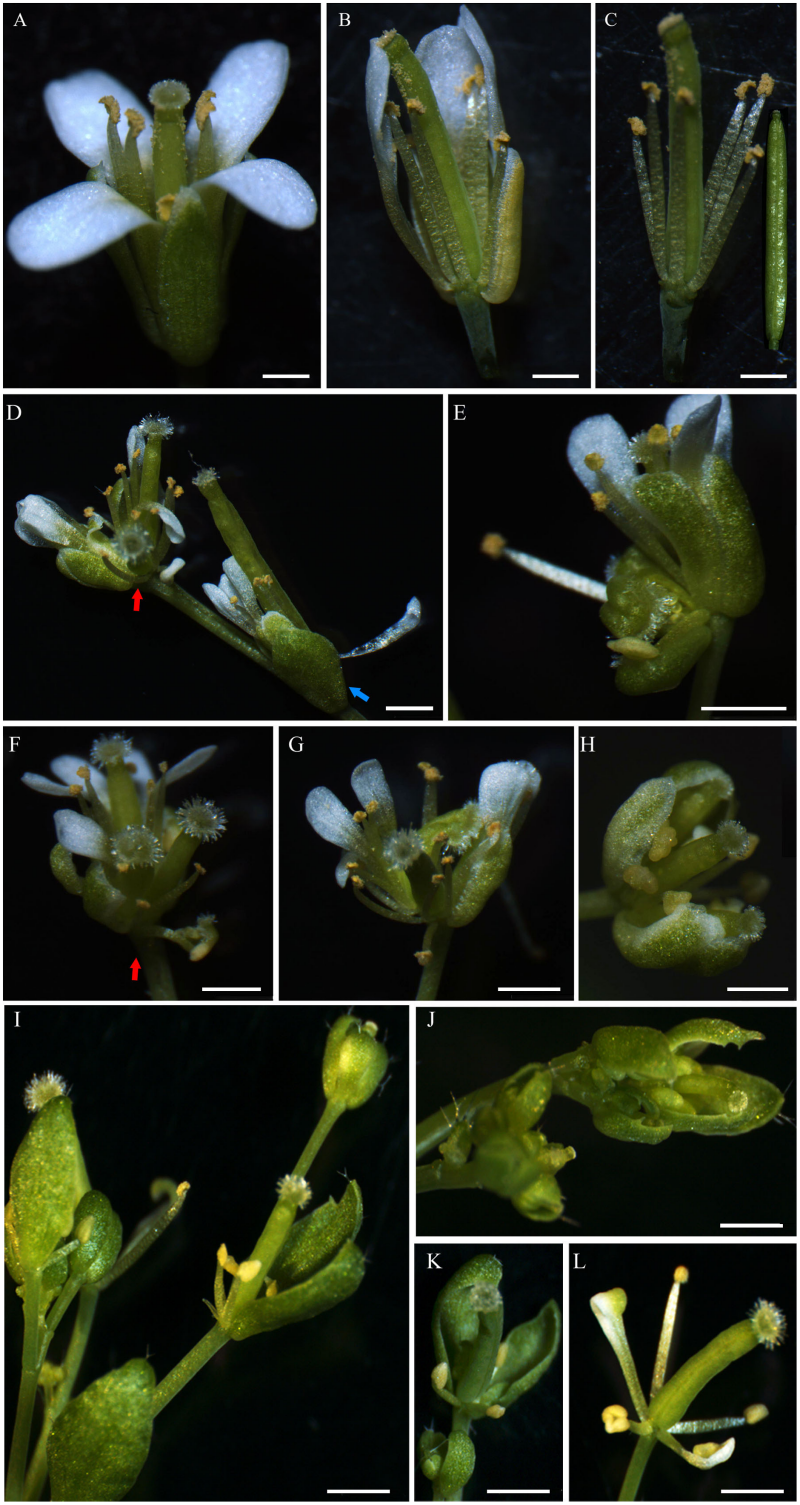
Abnormal phenotypes of various floral organs induced by *IiSEP2* overexpression. (A–C) The flowers of wild-type Col-0 Arabidopsis plants. The sepals and petals have been removed from Arabidopsis flower in (C). (D) *IiSEP2* transgenic line 3, red arrow indicate terminal flower containing a secondary flower, blue arrow indicate lateral flower. (E) *IiSEP2* transgenic line 7, the sepals were converted into carpelloid tissues. (F) *IiSEP2* transgenic line 4, red arrow indicate terminal flower containing a secondary flower. (G) *IiSEP2* transgenic line 5, five petals could be observed. (H) *IiSEP2* transgenic line 6, apetalous flower was generated. (I) Terminal flowers and secondary flower in crossing progeny of *IiSEP2* transgenic line 8 and wild-type Col-0. (J) *IiSEP2* transgenic line 8, abnormal flowers were produced. (K) Flower lacking petal in crossing progeny of *IiSEP2* transgenic line 8 and wild-type Col-0 plant. (L) Abnormal floral organs in crossing progeny of *IiSEP2* transgenic line 8 and wild-type Col-0 plant. Scale bars represent 1 mm.

Under normal circumstances, the flowers of the wild-type Col-0 Arabidopsis plants consist of four whorls of floral organs, including four sepals in the outermost whorl, four petals in the second whorl, six stamens in the third whorl, and a gynoecium in the fourth whorl (Figs. 5A–5C). By observation under stereomicroscope, it was found that overexpression of *IiSEP2* in Arabidopsis resulted in seriously anomalous development of the floral organs. A number of flowers were male sterile (Fig. 4D), owing to the abnormality of stamens in development. At the same time, the inflorescence meristems were transformed into defective terminal flowers without sepals and petals, and the number of stamens was reduced (Figs. 4E–4G). In Fig. 4E, only three stamens could be observed under the pistil in the middle. In many flowers, the sepals were converted homeotically into carpelloid structures with stigmatic papillae and ovules in transgenic lines 7 and 8 (Figs. 5E and 5J).

In transgenic lines 3 and 4, secondary flower could be generated in terminal flowers. The number of sepals, petals and stamens was decreased in these terminal flowers, whereas the secondary flowers were nearly normal in structure (Figs. 5D and 5F). Different from four petals in wild-type flowers, a few apical flowers of the *IiSEP2* overexpressing plants produced five petals or lacked petal, together with anomalous development of the carpels in transgenic lines 5 and 6 (Figs. 5G and 5H). Quantitative expression analyses showed that *AP3* and *PI*, two Arabidopsis MADS-box genes related to petal formation, were suppressed by ectopic expression of *IiSEP2* (Fig. S6). On the contrary, the transcripts of *SHP1* and *SHP2* were increased in *IiSEP2* transgenic Arabidopsis plants.

In order to fully understand the function of *IiSEP2*, transgenic line 8 was genetically crossed with the wild-type Col-0 plants, and the phenotypes of the crossing progenies were compared to that of the *IiSEP2* transgenic line 8. The phenotypes of the *IiSEP2* transgenic lines were maintained in filial generations of the crossing lines. These offsprings exhibited the phenotypic variations of the *IiSEP2* overexpressing plants, including the secondary flowers, the loss of petals, and the reduction of stamens (Figs. 5I, 5K, and 5L). All these phenotypic changes indicated that constitutive expression of *IiSEP2* could affect the morphogenesis of the flowers.

### Ectopic expression of *IiSEP4* in L*er* genetic background can affect the development of floral organs

In order to analyze the regulatory function of *IiSEP4* in floral transition and flower development, transgenic Arabidopsis plants were prepared. The transcriptional levels of *IiSEP4* in transgenic plants were detected by qRT-PCR. The results showed that *IiSEP4* could express effectively in transgenic plants of wild-type Arabidopsis in L*er* genetic background, and in transgenic plants of *ap1 cal* double mutant in L*er* genetic background (Fig. S7).

**Figure 6:**
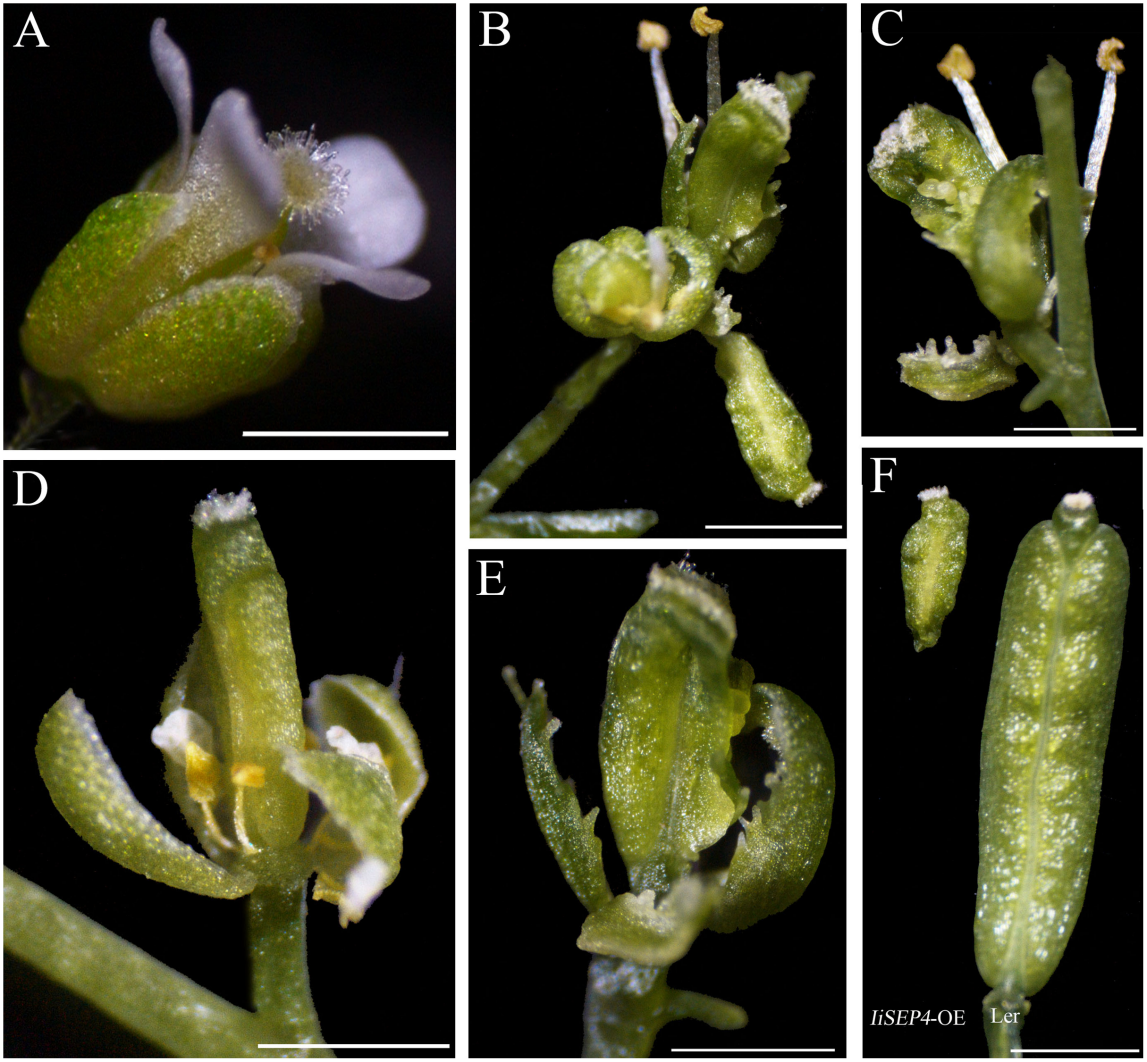
Phenotypic variations of the floral organs in *IiSEP4* transgenic Arabidopsis (in L*er* genetic background). (A) The flower of the wild-type L*er* plant. (B–E) The abnormal phenotype of the flowers in *35S::IiSEP4-GFP* transgenic plants. (F) The silique in *35S::IiSEP4-GFP* transgenic plant (left) and wild-type L*er* plant (right). Bar = 1 mm.

All the *35S::IiSEP4-GFP* transgenic plants displayed abnormal phenotypes, and the number of the floral organs was reduced. In particular, sepals were curly and were converted into carpelloid structures, and were obviously accompanied by the generation of ovules (Figs. 6B and 6C). In addition, dwarf and abnormal petals and stamens were produced (Fig. 6D), and the size of the silique was also decreased significantly in comparison with the wild-type L*er* plants (Fig. 6F).

**Figure 7:**
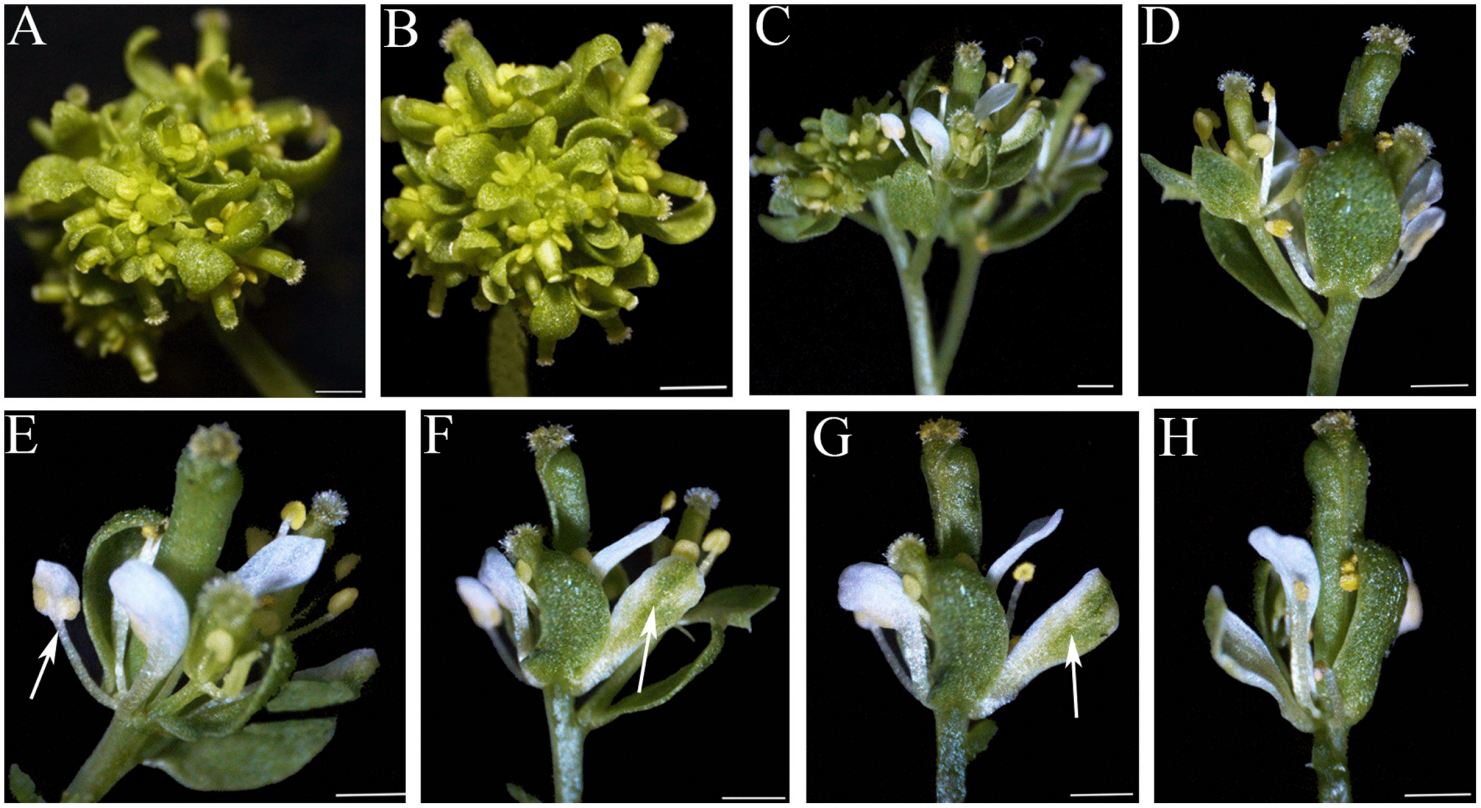
Phenotypic variations of *IiSEP4* transgenic plants of *ap1 cal* double mutant in L*er* genetic background. (A & B) The inflorescence of *ap1 cal* double mutant. (C–H) The inflorescence of *IiSEP4* transgenic plants of *ap1 cal* double mutant. (C) shows an incomplete cauliflower phenotype and petals reappeared. (D) is an enlarged photo of (C), a number of sepals, stamens and pistils can be found in one flower; In comparision with *ap1 cal* double mutant, multiple petals can be produced. (E) is the photo of the flower in (D) taken in different direction, arrow shows a chimeric organ composed of petaloid and stamineous tissues, representing fusion of petal and stamen. In (F and G), arrows show the chimeric organ composed of petaloid and sepaloid tissues, representing fusion of petal and sepal. (G and H) are the photos of the flower in (F) taken in different directions. Bar = 1 mm.

On account of the apetalous phenotype of *ap1 cal* double mutant, ectopic expression in this mutational material was conducted to clarify the roles of *IiSEP4* in petal differentiation. In *ap1 cal* double mutant in L*er* genetic background, floral meristems were transformed into inflorescence meristems, and new meristems were elaborated continuously. As a result, the inflorescence resembles a cauliflower in *ap1 cal* double mutant. In addition, *ap1 cal* plants produced apetalous flowers, namely only sepals, stamens and pistil could be formed (Figs. 7A and 7B). In *35S::IiSEP4-GFP* transgenic plants of *ap1 cal* double mutant, the cauliflower phenotype was attenuated significantly, and the petals could be recovered (Figs. 7C–7H). Occasionally, chimeric organ composed of petaloid and sepaloid tissues (Figs. 7F and 7G), and chimeric organ composed of petaloid and stamineous tissues (Fig. 7E), could be observed.

### Phenotypic variations of woad plants after downregulation of *IiSEP4* by VIGS

To analyze the moderating effects on woad flowering and to verify the results obtained in Arabidopsis, the expression level of *IiSEP4* was downregulated by VIGS mediated by *Agrobacterium*. The results of RT-PCR showed that RNA molecules of TRV1 and TRV2 could be detected in distal noninfiltrated leaves of the control woad plants treated with pTRV1 + pTRV2, and in distal noninfiltrated leaves of the woad plants treated with pTRV1 + pTRV2-IiSEP4 (Fig. S8). qRT-PCR data showed that the mRNA abundance of *IiSEP4* was reduced dramatically in woad plants treated with pTRV1 + pTRV2-IiSEP4 (Fig. S9), indicating *IiSEP4* could be silenced effectively by VIGS.

Silencing of *IiSEP4* can delay the flowering time. Compared to woad plants infiltrated with a mixture of *A. tumefaciens* GV3101 carrying pTRV1 and *A. tumefaciens* GV3101 carrying the empty pTRV2, the woad plants infiltrated with a mixture of *A. tumefaciens* GV3101 carrying pTRV1 and *A. tumefaciens* GV3101 carrying pTRV2-IiSEP4 start bolting nearly half a month later, indicating downregulation of *IiSEP4* can lead to late-flowering phenotype in woad. Specifically, wild-type woad plants begin to bloom in first ten-days of May, whereas *IiSEP4*-silenced lines bear flowers in late May.

**Figure 8:**
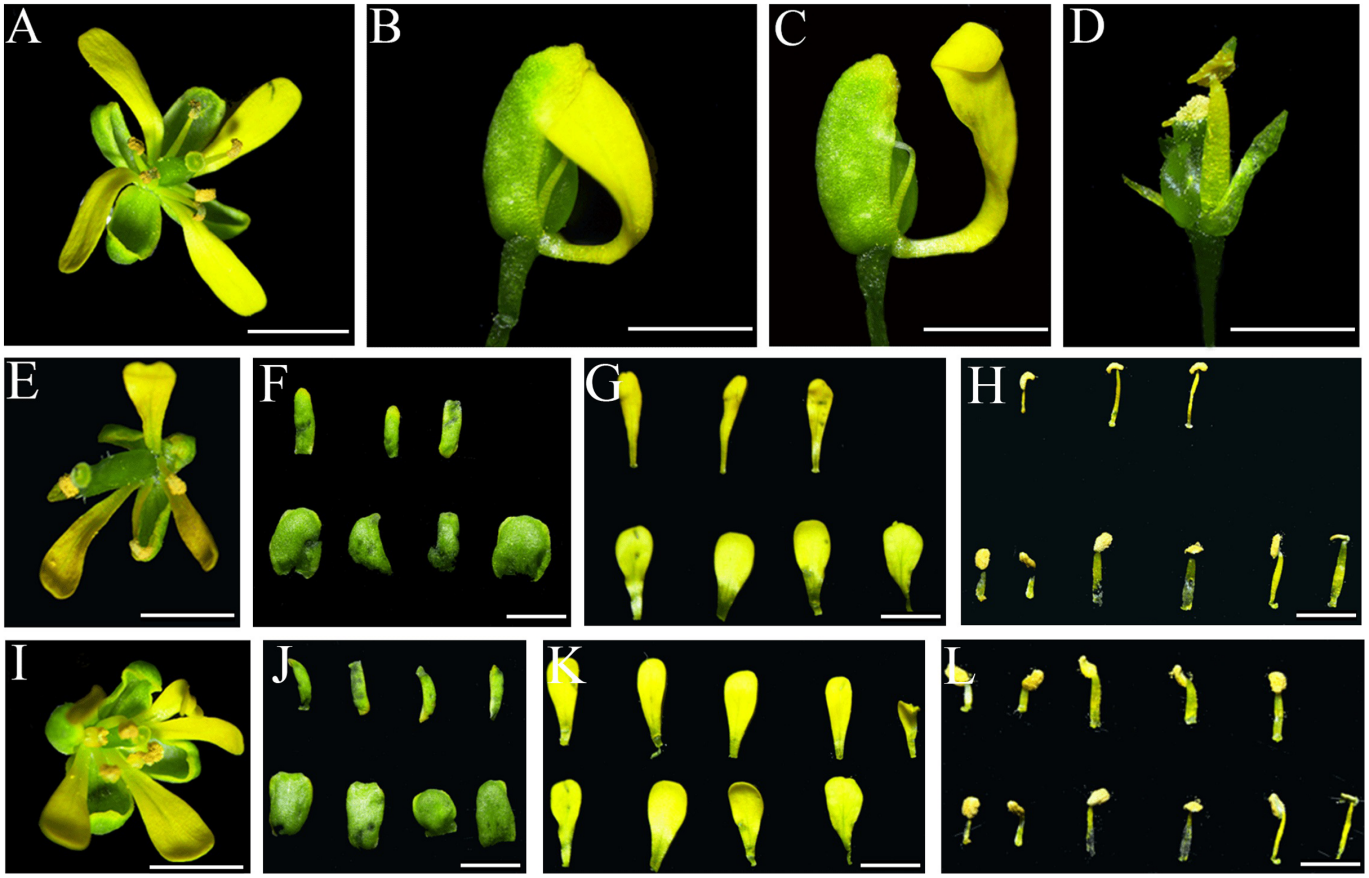
Phenotypic observation of the floral organs in woad plants treated with TRV-*IiSEP4*. (A) Wild-type woad flower. (B–L) Phenotypic variations of the flowers in woad plants infiltrated with TRV-*IiSEP4*. (B) a flower constituted by one sepal, one petal, one stamen and one pistil. (C and D) anatomy view of the flower in (B). (E) a flower with three sepals, three petals, three stamens and one pistil. (F–H) comparison of the size of the floral organs in the flower of (E) (up) with the floral organs in wild-type flower (down). (I) a flower contains four sepals, five petals, five stamens and one pistil. (J–L) comparison of the size of the floral organs in the flower of (I) (up) with the floral organs in wild-type flower (down). Bar = 1 mm.

More than 90% of the woad plants in treatment group showed phenotypic variations. Observation of the flowers showed that the floral organs were unchanged after infiltration with pTRV1 + pTRV2 (Fig. 8A), whereas the floral organs in woad plants infiltrated with pTRV1 + pTRV2-IiSEP4 presented pronounced anomalous phenotypes, and the number of sepals, petals and stamens was reduced (Figs. 8B–8E). In comparison with the control group, the size of the floral organs in woad plants infiltrated with pTRV1 + pTRV2-IiSEP4 was much smaller (Figs. 8F–8H). In some woad plants treated with pTRV1 + pTRV2-IiSEP4, the number of petals was increased. For instance, one flower in a woad plant infiltrated with pTRV1 + pTRV2-IiSEP4 produced five petals, but the number of stamens was reduced from six to five. In these plants, except the smaller sepals, no obvious change could be observed in the size of petals and stamens (Figs. 8I–8L).

## Discussion

Although A-, B- and C-class floral homeotic MADS-box genes are essential to the development of flowers, ectopic expression of these genes is not enough to completely induce the formation of floral organs, indicating other transcriptional factors are involved in floral patterning regulation (Krizek & Meyerowitz, 1996). Phenotypic observation of the multiple mutant showed that *SEPALLATA1/2/3/4* (*SEP1/2/3/4*) are also necessary for differentiation of the four types of floral organs, and for initiation of floral meristems (Pelaz et al., 2000; Ditta et al., 2004). Simultaneous mutation of *SEP1/2/3* converts the four types of floral organs into sepaloid tissues (Pelaz et al., 2000). If *SEP1/2/3/4* are all knocked out, the four types of floral organs will be reversed as leaf-like tissues bearing epidermal trichomes, representing the functions of the E-class floral homeotic genes are completely destroyed (Ditta et al., 2004).

Flower development of Arabidopsis from initiation until the opening was divided into 12 stages (Smyth, Bowman & Meyerowitz, 1990). *SEP2* was expressed in the entire floral meristems at stage 2, slightly earlier than *SEP3*. In subsequent stages, *SEP2* was expressed in all the four types of floral organs (Pelaz et al., 2000; Ditta et al., 2004). *ThtSEP2*, a *SEP2* homologous gene characterized in *T. thalictroides*, was expressed at low levels in all the floral organs and at all developmental stages of flowers (Soza et al., 2016). In the present work, the coding sequence of *IiSEP2* was cloned by degenerate PCR and its length is 753 bp, and the MADS-box protein encoded by *IiSEP2* consists of 250 amino acid residues. Multiple alignments showed that MADS-box proteins were highly conserved and the identity between IiSEP2 and Arabidopsis SEP2 was 95%. According to the results of qRT-PCR, *IiSEP2* was expressed in early stage of flower development, all the four types of floral organs, and silicles. In four types of floral organs, *IiSEP2* was highly expressed in sepals and petals. On the contrary, *IiSEP2* mRNA could not be detected obviously in vegetative tissues. These results illuminate that *IiSEP2* plays an ubiquitous and crucial role in flower development of *I. indigotica*.

In Arabidopsis, *SEP1* and *SEP2* are target genes of FAR-RED ELONGATED HYPOCOTYL3 (FHY3). FHY3 is associated with maintenance of shoot apical meristem, determinacy of floral meristem, and differentiation of petals and stamens. *SEP2* can be activated directly by FHY3, and ultimately promote the determinacy of floral meristem (Li et al., 2016). In *fhy3* single mutant, the pistil from the indeterminate floral meristem could generate plenty of floral organs. In inflorescences of *fhy3-68 ag-10* double *mutant*, the transcripts of *SEP1* and *SEP2* were decreased. On the contrary, *SEP3* expression was upregulated in *fhy3-68* single mutant and unchanged in *fhy3-68 ag-10* double mutant (Li et al., 2016). In consistent with this, ectopic expression of *IiSEP2* in Arabidopsis can also influence the normal development of the floral organs and the siliques. Similar to *35S::IiSEP4-GFP* transgenic plants in wild-type L*er* genetic background obtained in the present work (Fig. 6F), *FHY3* mutants in the genetic background of *ag-10* produced very short and bulged siliques with an increased carpel number, accompanied by formation of very small petals and sterile anthers. *IiSEP2* and *IiSEP4* of *I. indigotica* probably also play an important role in determinacy of the floral meristems, and are controlled by the ortholog of FHY3 in *I. indigotica*.

Ectopic expression of *SEP2* could rescue the defects of *fhy3-68 ag-10* double mutant in floral meristem determinacy. In *35S:SEP2 fhy3-68 ag-10* transgenic Arabidopsis plants, normal siliques composed of gynophore and two fused carpels could be produced. It suggested that the function of *FHY3* in floral meristem determinacy was mediated by *SEP2*. However, the other phenotypic variations in *fhy3-68 ag-10* double mutant, including small inflorescence, short petal and sterile anther, could not be rescued by *SEP2*. Different from *SEP2*, overexpression of *SEP1* in *fhy3-68 ag-10* double mutant under the control of CaMV 35S promoter could not recover the defective phenotypes of floral meristems, indicating *SEP2* possesses an unique role in pistil development and in determinacy of flowers. When *SEP2* was driven by the flower-specific *SEP3* promoter, its expression in *fhy3-68 ag-10* double mutant could also rescue the floral meristem indeterminacy. Nonetheless, short siliques with multiple carpels and additional organs, and sterile anthers could be produced. When *SEP2* was down-regulated by artificial miRNA in the genetic background of *ag-10*, bulged siliques with additional organs growing inside were generated, similar to the phenotype of *fhy3-68 ag-10* double mutant, indicating suppression of *SEP2* can enhance the indeterminacy of floral meristem (Li et al., 2016). *IiSEP2* and *IiSEP4* may also possess divergent or specified functions in comparison with other *SEP*-like genes in *I. indigotica*.

*SEP*-like genes were found to be involved in regulation of the inflorescence architecture. In *sep1sep2sep3* triple mutant and *sep1sep2sep3sep4* quadruple mutant, the degree of inflorescence branching was increased (Pelaz et al., 2000; Ditta et al., 2004). The transcript of *SlCMB1*, a *SEP*-like gene of tomato, was mainly accumulated in sepals. In RNA interference lines of *SlCMB1*, longer peduncles and more branches were generated. Moreover, generation of new leaves and apical meristem in RNA interference lines represents a loss of the inflorescence determinacy and a transition from reproductive growth to vegetative growth. In particular, abnormally fused and enlarged sepals could be observed in RNA interference lines (Zhang et al., 2018). In *Gerbera hybrida*, *SEPALLATA*-like MADS-box genes were associated with patterning regulation of the pseudanthial inflorescence (Zhang et al., 2017b). In monocotyledons, *SEPALLATA*-like genes could also influence the architecture of inflorescences (Gao et al., 2010; Lin et al., 2014; Song et al., 2018). In the present work, it was confirmed that overexpression of *IiSEP2* and *IiSEP4* in Arabidopsis resulted in reduction of the flower number and the lateral branch number.

*TM5* is a *SEP*-like gene in tomato and is expressed at high levels in meristematic territories fated to form petals, stamens, and gynoecia. Suppression of *TM5* resulted in morphogenetic alterations in the internal three whorls of floral organs. In antisense transgenic plants of *TM5*, the petals were green throughout the life span of the flowers, the anthers were also green and were converted into sepaloid or petaloid structures. Moreover, incomplete fusion of carpels and failure to form normal style could be observed (Pnueli et al., 1994). As a *SEP*-like gene in rice, severe loss-of-function mutations in *OsMADS1* could cause complete homeotic conversion of lodicules, stamens and carpels into lemma-like or palea-like structures (Agrawal et al., 2005). *PlacSEP1.1*, *PlacSEP1.2* and *PlacSEP1.3* of London plane have been characterized functionally. Overexpression of *PlacSEP1.1*, *PlacSEP1.2* and *PlacSEP1.3* in *Arabidopsis* resulted in different phenotypic alterations. *35S:PlacSEP1.1* transgenic plants with the small size and curled leaves were early flowering obviously. *35S:PlacSEP1.3* transgenic lines also exhibited early flowering phenotype, and possessed less rosette leaves and more cauline leaves in comparison with the wild-type *Arabidopsis* plants. However, curled leaf could not be found. On the contrary, *35S:PlacSEP1.2* transgenic plants showed no visible phenotypic changes (Zhang et al., 2017a). *TaMADS1* is a *SEP*-like gene isolated from wheat (*Triticum aestivum* L.). Compared to wild-type *Arabidopsis*, *35S:TaMADS1* transgenic lines were early flowering, the number of petals and stamens was reduced, some flowers had short filament and sterile petaloid anthers (Zhao, Cheng & Zhang, 2006). All these reports together with the results in the present work indicated that *SEP*-like genes were conserved in regulation of the development of floral organs in both dicotyledons and monocotyledons.

In this work, influence of *IiSEP2* overexpression on MADS-box genes of Arabidopsis was also analyzed, including *AP3*, *PI*, *SHP1* and *SHP2*. *AP3* and *PI* are B-class floral homeotic MADS-box genes and are responsible for specifying the identity of petals. *SHP1* and *SHP2* are associated with formation of the dehiscence zone in siliques and development of ovules (Pinyopich et al., 2003). The results showed that *AP3* and *PI* were downregulated in *IiSEP2* transgenic Arabidopsis plants, whereas *SHP1* and *SHP2* were upregulated. Similar to *IiSEP2* and *IiSEP4*, constitutive expression of *SHP* in Arabidopsis also resulted in ectopic formation of carpels and ovules (Favaro et al., 2003). In like manner, when *LMADS2*, a carpel-specific MADS-box gene of *Lilium longiflorum*, was constitutively expressed in Arabidopsis, sepals would be converted homeotically into carpelloid structures with stigmatic papillae and ovules (Tzeng, Chen & Yang, 2002).

Moreover, the apical flowers in *IiSEP2* transgenic Arabidopsis plants often produced five petals, three pistils, shrunken stamens and secondary flowers, or apetalous. These data showed that the development of petals and stamens in Arabidopsis could be affected by ectopic expression of *SEP*-like genes, and phenotypic changes similar to *ap3-3* and *pi-1* mutants could be produced (Bowman et al., 1999). Quantitative real-time PCR showed that the expression of *AP3* and *PI* could be inhibited by IiSEP2. However, ectopic expression of *IiSEP4* could restore the development of petals in *ap1 cal* double mutant.

Except for the influences on flower development, *IiSEP2* and *IiSEP4* can affect the growth of leaves. The cauline leaves in *IiSEP2* transgenic Arabidopsis are shaped like inverted cones, and the angles between the cauline leaves and the stems were reduced, which was probably associated with *CURLY LEAF* (*CLF*). The protein encoded by *CLF* was a subunit of polycomb repressive complex 2 (PRC2). CLF could change the transcriptional activity of the target genes by trimethylation of lysine 27 on histone H3 (H3K27me3) with its methyltransferase activity, and resulted in repression of the target genes (Ré et al., 2020; Singkaravanit-Ogawa et al., 2021). *clf* mutants showed leaf curling and early flowering phenotypes, which could be suppressed by mutation of *SEP3*. It has been reported that derepression of *SEP3* could cause alterations in morphology of leaves (Mateo-Bonmatí et al., 2018). In *clf* mutant, *SEP3* was highly expressed in leaves. Consistent with this, ectopic expression of *SEP3* driven by CaMV 35S promoter produced curled rosette leaves (Pelaz et al., 2001), and overexpression of *SEP*-like genes can also alter the morphology of leaves (Adal et al., 2021).

## Conclusions

In Arabidopsis, ectopic expression of *IiSEP2* could lead to early flowering. In woad, downregulation of *IiSEP4* by VIGS could produce late-flowering phenotypes. In comparison with the wild-type plants, transgenic Arabidopsis plants of *IiSEP2* and *IiSEP4* were relatively small and the main stems of these plants were thin. Constitutive expression of *IiSEP2* and *IiSEP4* in Arabidopsis could disturb the indeterminacy of inflorescence meristems, and only a small number of flower buds would be produced, accompanied by generation of terminal flower. Furthermore, *IiSEP2* and *IiSEP4* can influence the development of floral organs. In transgenic plants overexpressing *IiSEP2* and *IiSEP4*, abnormal floral structures without sepals and petals were formed, and the number of stamens was reduced. Meanwhile, carpelloid structures with stigmatic papillae and ovules could be observed. In *IiSEP4* transgenic plants in the genetic background of *ap1 cal* double mutant, petal formation could be rescued.

## Supplemental Information

10.7717/peerj.13034/supp-1 Supplemental Information 1 Supplementary Table, Figures and Sequence data.

10.7717/peerj.13034/supp-2 Supplemental Information 2 Raw Data and original gel images-revision.

## Additional Information and Declarations

### Data Availability

The following information was supplied regarding data availability: The raw data and full-length gel images are available in the Supplemental Files.
