# Supplementary material for: SEPALLATA­-like genes of Isatis indigotica can affect the architecture of the inflorescences and the development of the floral organs
Source: PeerJ. 2022 Mar 1;10:e13034. doi: 10.7717/peerj.13034 (PMC8896020; doi:10.7717/peerj.13034)
Supplement: Supplemental Information 1 [file peerj-10-13034-s001.doc]

| **Table S1 Primers used in vector construction and expression analyses.**  Primer name Sequence (5'→3') | |
| --- | --- |
| IiSEP2-OE-up | GCGTCTAGAATGGGAAGGGGAAGAGTAGAG |
| IiSEP2-OE-down | GCGGAGCTCCAGAGCATCCAGCCTGGGATG |
| IiSEP2-GFP-FPrimer | GCGCCATGGGAAGGGGAAGAGTAGAG |
| IiSEP2-GFP-RPrimer | GCGACTAGTGAGCATCCAGCCTGGGATGT |
| IiSEP2-Upstream1 | CCACGGTTGGAGAAGACGATGAGGGAAACC |
| IiSEP2-Upstream2 | CGGCATCGCAGAGAACAGAAAGCTCATAAG |
| IiSEP2-qRT-F | AGACGGCTCTCTGAAGCAAG |
| IiSEP2-qRT-R | TATGGTGACTTCTCACGCCG |
| IactinF | TATTGTTGGTCGTCCCAGGC |
| IactinR | ACGACCACTGGCGTAAAGAG |


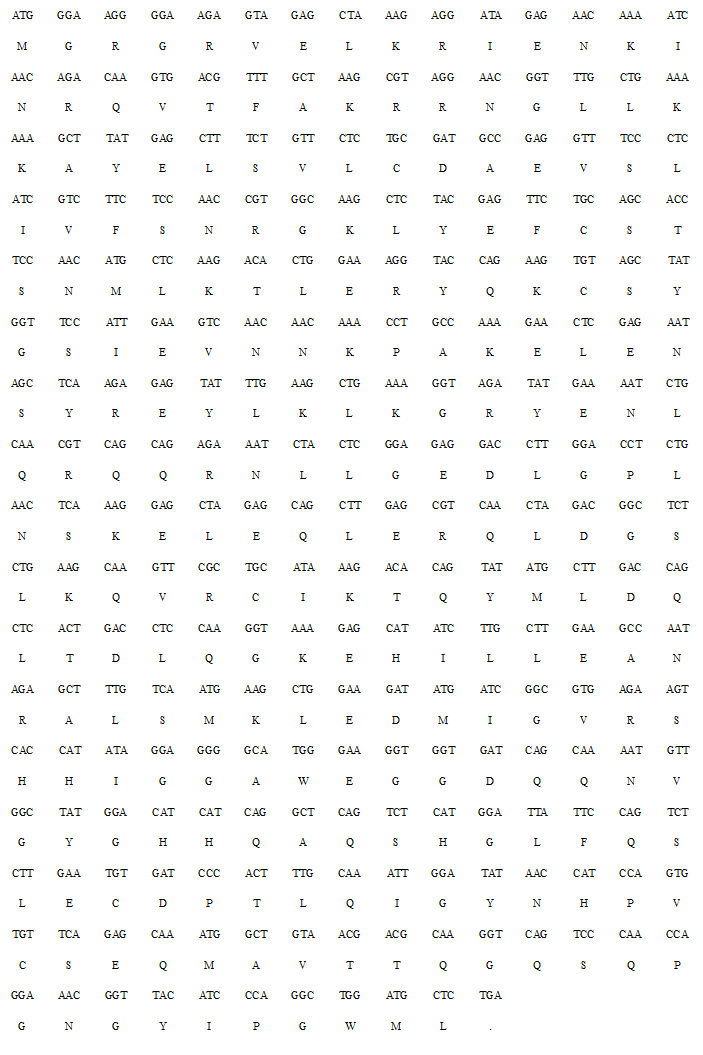
**Figure S1 The coding sequence of *IiSEP2* and the amino acid sequence of the encoded product.**


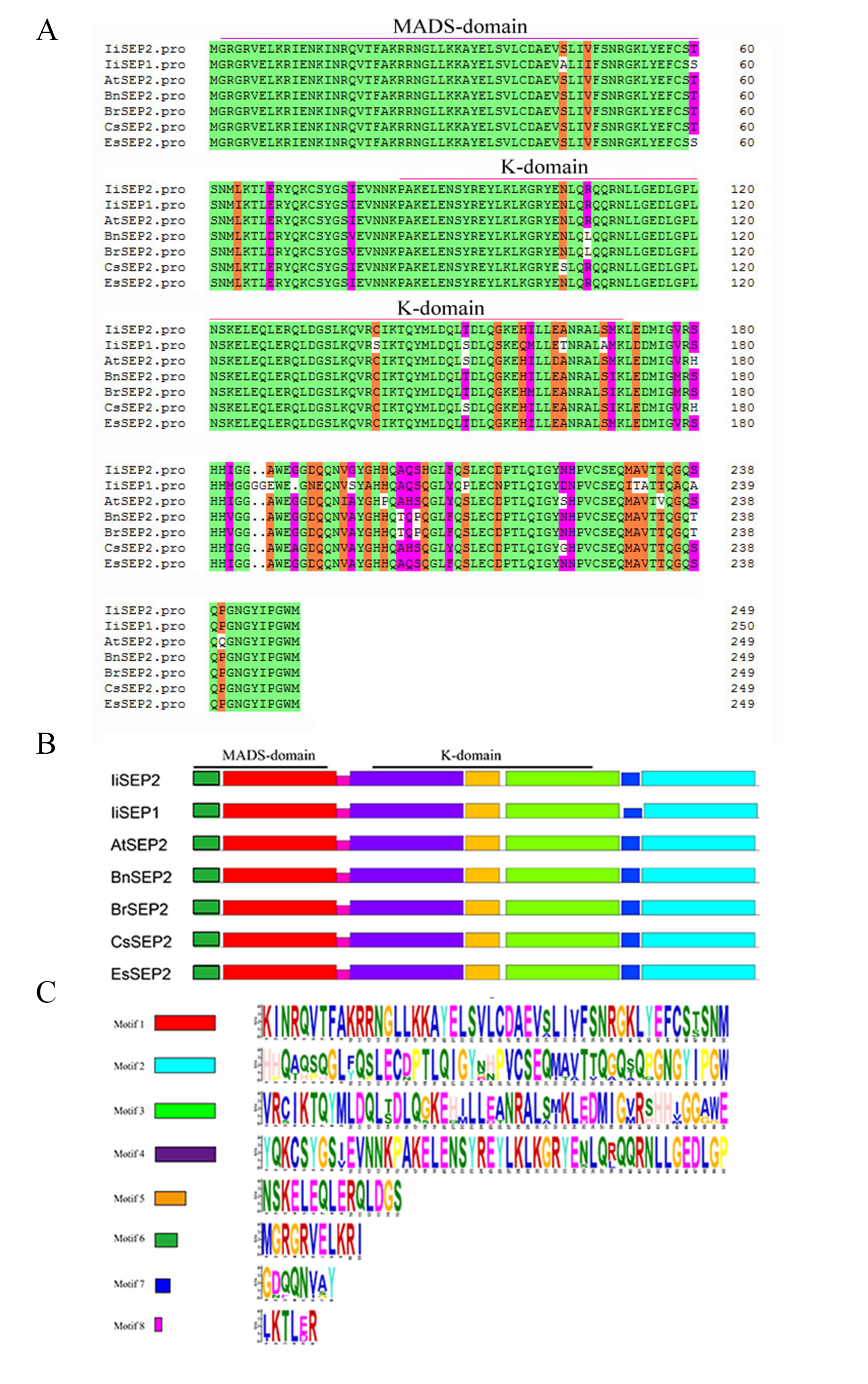
**Figure S2 Comparison of the structure of SEP2 homologous proteins.** (A) Alignment of the amino acid sequences of SEP2 homologous proteins. Green indicates the identical amino acid, pink and orange indicate the different amino acid. AtSEP2, *Arabidopsis thaliana* SEP2 (NM_111098.4); EsSEP2, *Eutrema salsugineum* SEP2 (XM_006408363.2); BnSEP2, *Brassica napus* SEP2 (XM_013822948.2); BrSEP2, *Brassica rapa* SEP2 (XM_009149111.2); CsSEP2, *Camelina sativa* SEP2 (XM_010487167.1); IiSEP1, *Isatis indigotica* SEP1 (BBJ25277.1). (B) Motif analysis. (C) The sequence logo for motifs. The height of the letters represents the degree of conservativeness.

**
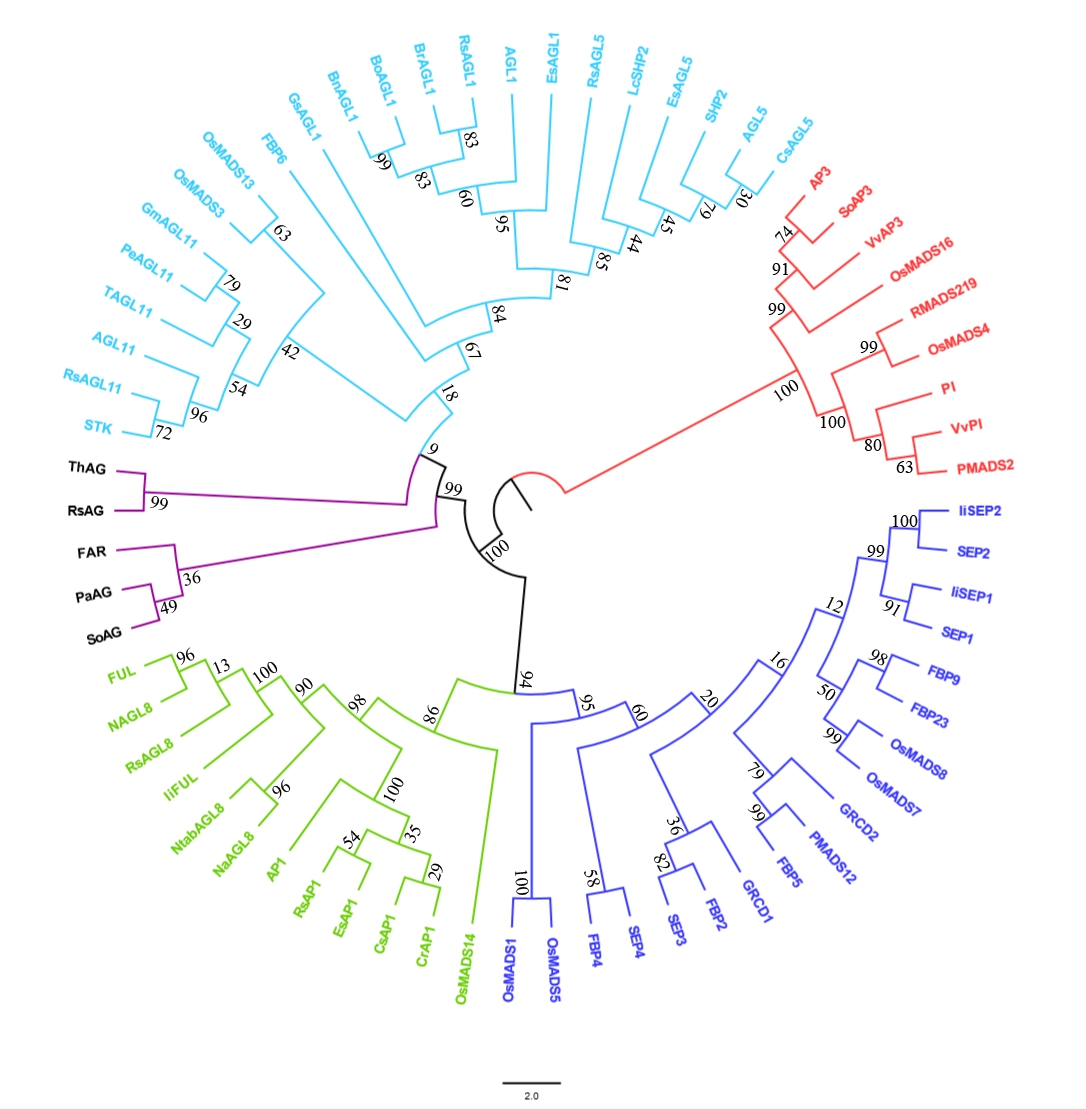
 Figure S3 Phylogenetic analysis based on multiple alignment of the amino acid sequences of plant MADS-box proteins.** E clade was shown in blue. AGL1, AAA32730.1; AGL11, NP_001078364.1; AGL12, AAC49085.1; AGL13, NP_191671.1; AGL5, AAA32735.1; AGL8, NP_568929.1; AP1, NP_177074.1; AP3, NP_191002.1; BnAGL1, XP_022573522.1; BoAGL1, XP_013605337.1; BrAGL1, XP_009116562.1; CrAP1, XP_006301567.1; CsAGL5, XP_010506124.1; CsAP1, XP_010511937.1; EsAGL1, XP_024012820.1; EsAGL5, XP_024016862.1; EsAP1, XP_006391056.1; FAR, BAI68392.1; FBP2, AAA86854.1; FBP23, AAK21254.1; FBP4, AAK21247.1; FBP5, AAK21248.1; FBP6, CAA48635.1; FBP9, AAK21249.1; FUL, NP_568929.1; GmAGL11, NP_001236130.1; GRCD1, CAC13148.1; GRCD2, CAH04878.1; GmAGL1, NP-001240100.1; IiFUL, BBB04554.1; IiSEP1, BBJ25277.1; LcSHP2, CBY05405.1; NaAGL8, XP_019238190.1; NtabAGL8, NP_001312873.1; OsMADS1, XP_015628585.1; OsMADS13, AAF13594.1; OsMADS14, XP_015631034.1; OsMADS16, XP_015641661.1; OsMADS3, XP_015632490.1; OsMADS4, Q40703.3; OsMADS5, AAB71434.1; OsMADS7, AAC49816.2; OsMADS8, Q9SAR1.1; PmAG, XP_008225407.1; PeAGL11, XP_011031534.1; PI, NP_197524.1; PMADS2, CAA49568; RsAG: XP_018468203.1; RsAGL1, XP_018446598.1; RsAGL11, XP_018452528.1; RsAGL5, XP_018437427; RsAGL8, XP_018436529.1; RsAP1, XP_018468274.1; SEP1, NP_001119230.1; SEP2, NP_186880.1; SEP3, NP_564214.2; SEP4, NP_178466.1; SHP2, NP_850377.1; SoAG, XP_021845998.1; SoAP3, ADD14338.1; STK, NP_001329612.1; TAGL11, XP_010312975.1; ThAG, XP_010538582.1; VvAP3, NP_001267960; VvPI, NP_001267875.

**
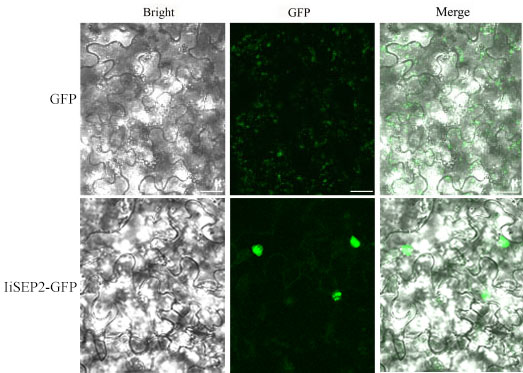
**

**Figure S4 Subcellular localization of IiSEP2.** The leaves of N. benthamiana were infiltrated with *Agrobacterium* cells carrying the constructs containing *IiSEP2-GFP* or *GFP* under the control of CaMV 35S promoter. Nuclear localization of IiSEP2 was confirmed by transient expression.


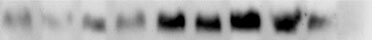

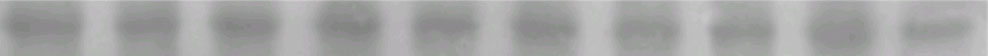


IiSEP2-GFP

Robisco

1 2 3 4 5 6 7 8 9 WT

**Figure S5 Identification of transgenic Arabidopsis plants using rabbit monoclonal antibody of GFP.** 1-9, independent transgenic Arabidopsis plants. WT, wild-type Arabidopsis plants.

**
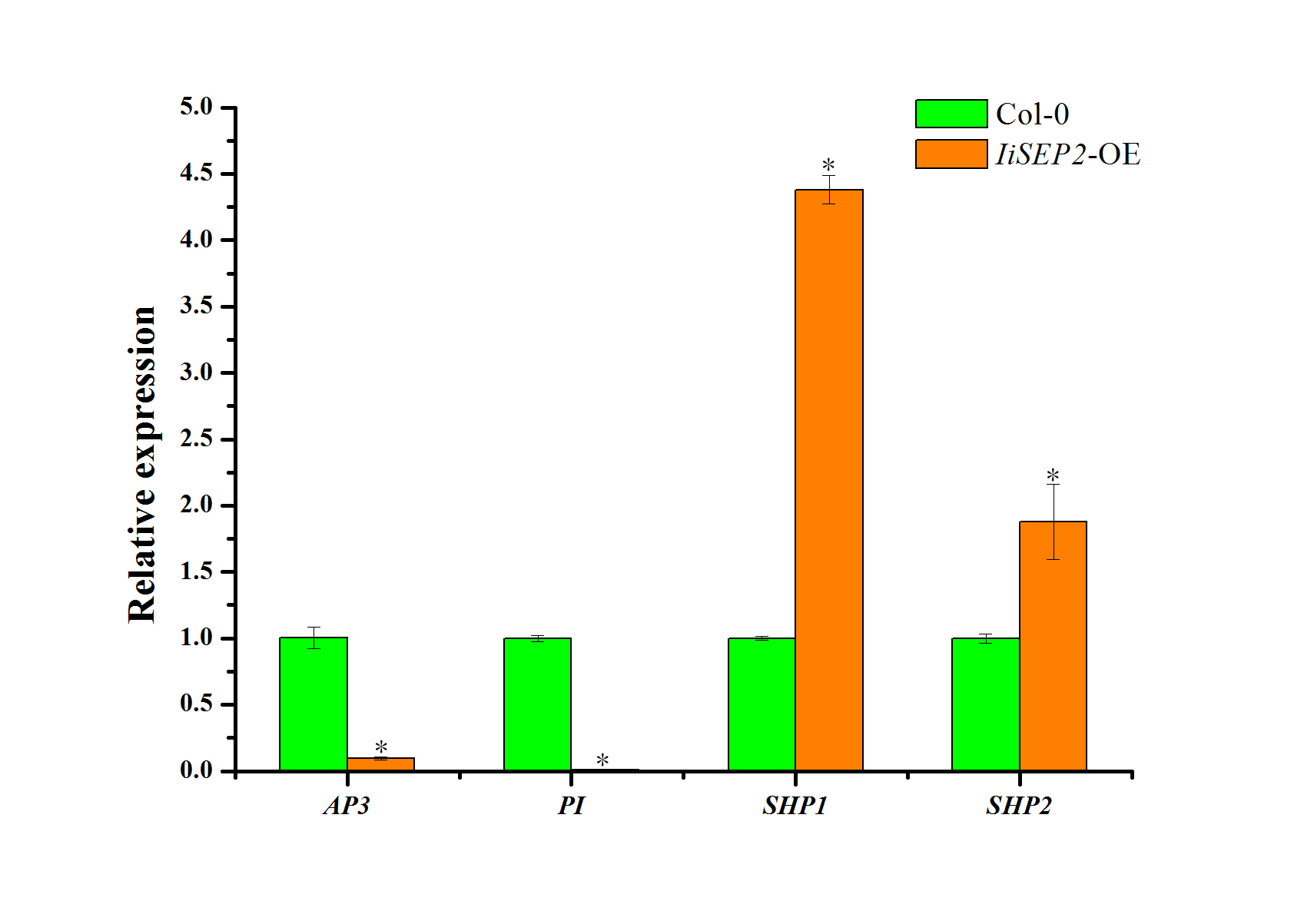
**

**Figure S6 qRT-PCR analysis of the MADS-box genes in Arabidopsis.** Error bars represent the standard deviation. Significant differences according to Student's *t*-test are indicated. *, P < 0.05.

**
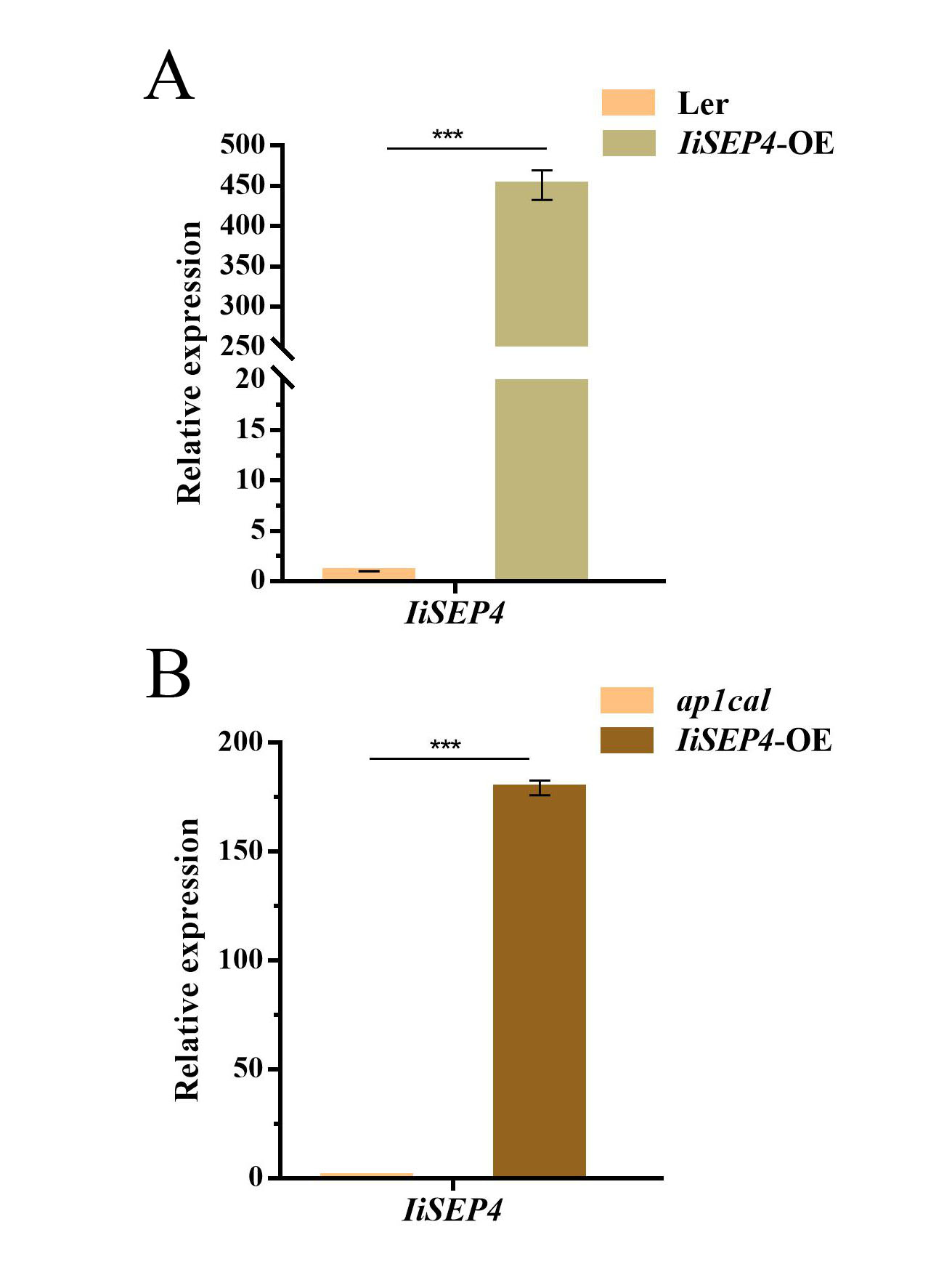
**

**Figure S7 qRT-PCR analysis of *IiSEP4* transgenic Arabidopsis.** (A) 35S::*IiSEP4-GFP* transgenic plants in wild-type Ler genetic background. (B) 35S::*IiSEP4-GFP* transgenic plants of *ap1 cal* double mutant in Ler genetic background. Error bar represents the standard deviation. Signiﬁcant diﬀerences according to Student's *t*-test are indicated. ***, P < 0.001.

**
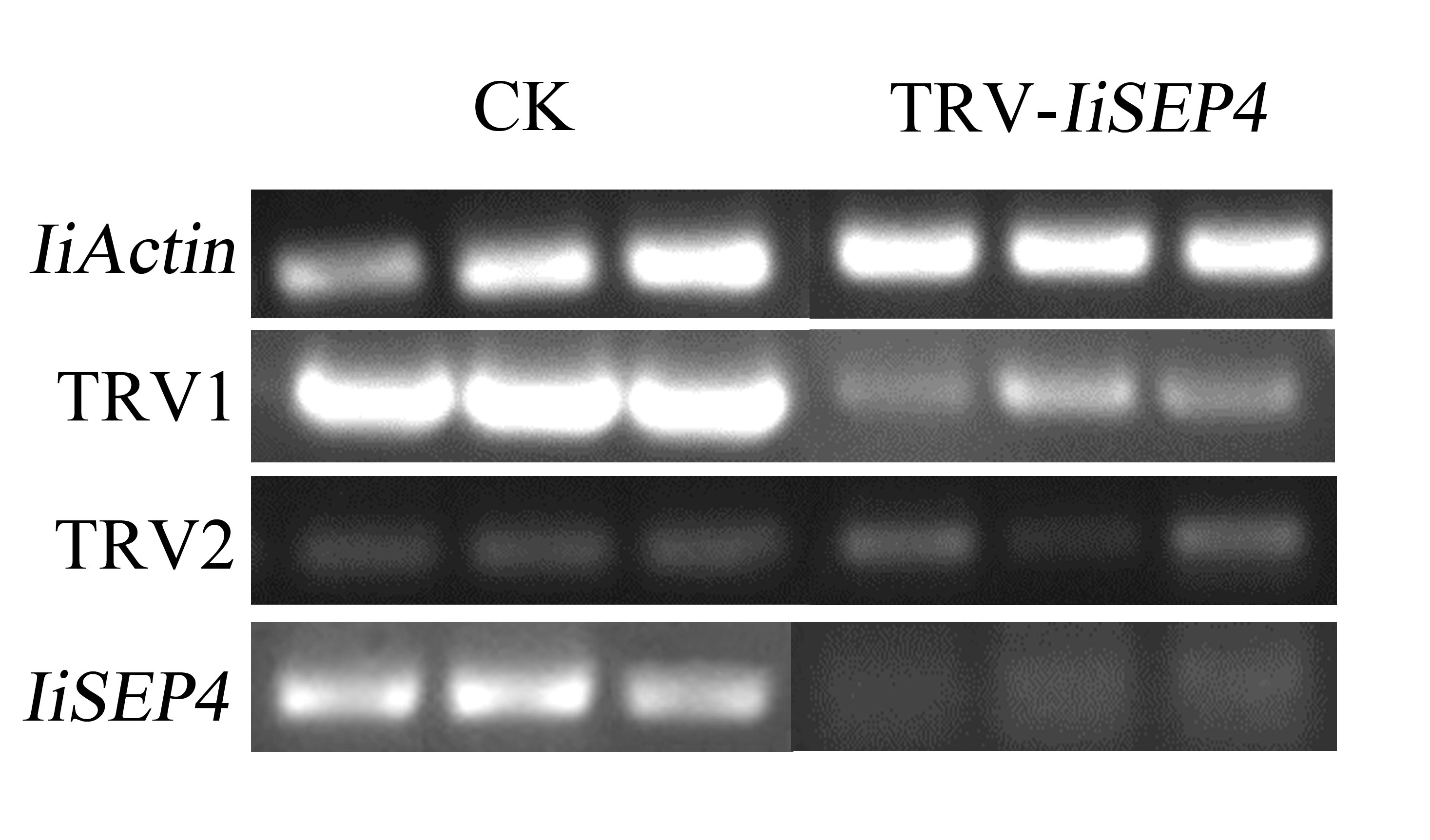
**

**Figure S8 RT-PCR analyses of TRV RNA and *IiSEP4* mRNA in distal noninfiltrated leaves.** CK, woad plants infiltrated with pTRV1 + pTRV2; TRV-*IiSEP4*, woad plants infiltrated with pTRV1 + pTRV2-IiSEP4. *IiActin* was used as a reference gene.

**
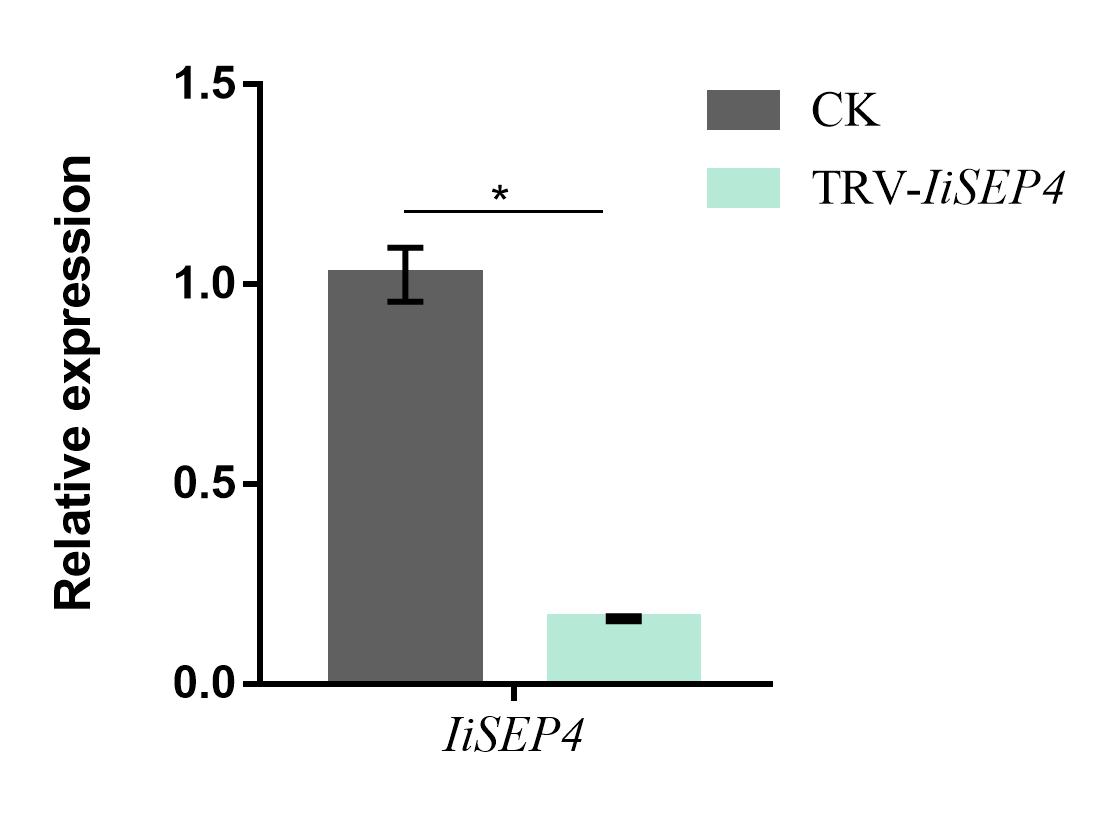
**

**Figure S9 qRT-PCR analyses of *IiSEP4* in distal noninfiltrated leaves.** CK, woad plants infiltrated with pTRV1 + pTRV2; TRV-*IiSEP4*, woad plants infiltrated with pTRV1 + pTRV2-IiSEP4.

**Supplementary sequence data 1 The sequence of IiSEP2 promoter.** Green shows the CArG-box. Yellow shows the initiation codon.

CTGGTGAAGTAGAAAAATGATGATGGCTGCTTTCGTATTATTGTAGAAGCCAATATTGTCCCCACTCTCACGCGCCTTACCACTTGGACAGTCCTTTTATATTTTCATTTCATTGGAACTTTTTTTTTATATATATCTAACTACATATATAACACACGTGCTTATTGTGTAATGATGAAACGATAACTCTCTTTGGCTCTAGTAATGTAATTTGAGCTGATGACAAATCAAAAGAAAAATTTCGGTATCACACCTTGAGCTAAAAAAAAAAAAAAAAGAGGTACGTACATTACAAATTGTAAAAACATTTGCATGTATAATAGAGTTACGCTATATCGATATCGTTGTTTCAAATATTTCTATGAAGTGAGAAAATGTATGTAAACCTAAAAGAGTGGAAACATAGTATCTAATTATCTATATATAGACCTGAATTATAATTAAAAGAAAAACAGAGACTTTATTTCTCTTAATTAGTAGCAACTCATAGCAAACAATGGTGTCTGTATGACACGCGGGAAGAAAAGAGAGTAGGAGGATGTTTTTTAACTTTTTTGTAAAAGAGGGGAGGACAGAGAAAGAGTAAAAAAGTAGAGAGAGAGTGTGAAACACACCAGATATATCACCAAACCCTAATAATCTCTCACCCTCACAAATTTCTTATCTCTATAGCTTTTATAGATTCACAAAAGCTTTCTTCAGATTCACAATCTCATCACACACACACAAGCCTTCAAAAAAAGAAAGAAAGAAAATAAAAGCAAACAAAAGATCTAAAGATTAATAACCAAGAACCCTACAATCAAATCAAAACCAAAATCAGAAAGCAAAAGTTTCCTCTTTTATTTTCTTCTCTCAATTCCTCTTCTTCCTGTTCTTGAAAAACTAGGGTTTTTATTGTATATTCTCCAAAAGAAAAGATCTTTCATCAGAAAAAGCAATACCCAACTCATGTCTCTGTGTGTCTGTATATAGATAAGCATTTACACACCCTAATAAGGTTACATATACACATATAAAAGAAGAAAGAAGTTAGGGATTTTGGGGAGAAATGGGAAGGGGAAGAGTAGAGCTAAAGAGGATAGAGAACAAAATCAACAGACAAGTGACGTTTGCTAAGCGTAGGAACGGTTTGCTGAAAAAAGCTTATGAGCTTTCTGTTCTCTGCGATGCCGAGGTTTCCCTCATCGTCTTCTCCAACCGTGGCAAGCTCTACGAGTTCTGCAGCACCTCC
